# *Ceratocystis cacaofunesta* genome analysis reveals a large expansion of extracellular phosphatidylinositol-specific phospholipase-C genes (PI-PLC)

**DOI:** 10.1186/s12864-018-4440-4

**Published:** 2018-01-17

**Authors:** Eddy Patricia Lopez Molano, Odalys García Cabrera, Juliana Jose, Leandro Costa do Nascimento, Marcelo Falsarella Carazzolle, Paulo José Pereira Lima Teixeira, Javier Correa Alvarez, Ricardo Augusto Tiburcio, Paulo Massanari Tokimatu Filho, Gustavo Machado Alvares de Lima, Rafael Victório Carvalho Guido, Thamy Lívia Ribeiro Corrêa, Adriana Franco Paes Leme, Piotr Mieczkowski, Gonçalo Amarante Guimarães Pereira

**Affiliations:** 1 Genomic and Expression Laboratory, Department of Genetics, Evolution and Bioagents, Institute of Biology, University of Campinas, Campinas, SP 13083-970 Brazil; 2 Centro Nacional de Processamento de Alto Desempenho, Universidade Estadual de Campinas, Campinas, Brazil; 3 Departamento de Ciencias Biológicas, Escuela de Ciencias, Universidad EAFIT, Medellın, Colombia; 4 Centro de Biotecnologia Molecular Estrutural, Instituto de Física de São Carlos, Universidade de São Paulo, São Paulo, Brazil; 5 Brazilian Biosciences National Laboratory (LNBio) CNPEM, Campinas, Brazil; 6 High-Throughput Sequencing Facility, University of North Carolina, Chapel Hill, NC USA; 7 Present Address: Department of Biology, University of North Carolina at Chapel Hill, Chapel Hill, NC 27599 USA

**Keywords:** Phosphoinositide-specific phospholipases C (PI PLC), *Ceratocystis* wilt of cacao, Plant pathogen

## Abstract

**Background:**

The *Ceratocystis* genus harbors a large number of phytopathogenic fungi that cause xylem parenchyma degradation and vascular destruction on a broad range of economically important plants. *Ceratocystis cacaofunesta* is a necrotrophic fungus responsible for lethal wilt disease in cacao. The aim of this work is to analyze the genome of *C. cacaofunesta* through a comparative approach with genomes of other Sordariomycetes in order to better understand the molecular basis of pathogenicity in the *Ceratocystis* genus.

**Results:**

We present an analysis of the *C. cacaofunesta* genome focusing on secreted proteins that might constitute pathogenicity factors. Comparative genome analyses among five *Ceratocystidaceae* species and 23 other Sordariomycetes fungi showed a strong reduction in gene content of the *Ceratocystis* genus. However, some gene families displayed a remarkable expansion, in particular, the Phosphatidylinositol specific phospholipases-C (PI-PLC) family. Also, evolutionary rate calculations suggest that the evolution process of this family was guided by positive selection. Interestingly, among the 82 PI-PLCs genes identified in the *C. cacaofunesta* genome, 70 genes encoding extracellular PI-PLCs are grouped in eight small scaffolds surrounded by transposon fragments and scars that could be involved in the rapid evolution of the PI-PLC family. Experimental secretome using LC–MS/MS validated 24% (86 proteins) of the total predicted secretome (342 proteins), including four PI-PLCs and other important pathogenicity factors.

**Conclusion:**

Analysis of the *Ceratocystis cacaofunesta* genome provides evidence that PI-PLCs may play a role in pathogenicity. Subsequent functional studies will be aimed at evaluating this hypothesis. The observed genetic arsenals, together with the analysis of the PI-PLC family shown in this work, reveal significant differences in the *Ceratocystis* genome compared to the classical vascular fungi, *Verticillium* and *Fusarium*. Altogether, our analyses provide new insights into the evolution and the molecular basis of plant pathogenicity.

**Electronic supplementary material:**

The online version of this article (10.1186/s12864-018-4440-4) contains supplementary material, which is available to authorized users.

## Background

*Ceratocystis cacaofunesta* (Phylum: Ascomycota; Class: Sordariomycetes) is the causal agent of *Ceratocystis* wilt of cacao (CWC), a disease responsible for significant losses suffered by the cacao industry in both Central and South America [1–3]. The genus *Ceratocystis* encompasses numerous plant pathogens, including the sweet potato pathogen *Ceratocystis fimbriata* [4, 5], the plane tree pathogen *Ceratocystis platani* [6] and the mango pathogen *Ceratocystis manginecans* [7]. Pathogens of *Ceratocystis* genus cause diverse diseases, such as root and tuber rot, canker stains and vascular wilt [8, 9] in a broad range of economically important crops around the world [5, 10]. CWC is a severe disease that begins with the fungus accessing the host tissue through wounds caused by beetles or by contaminated tools during pruning [11, 12]. Once inside the plant tissue, chlamydospores (aleurioconidia) germinate, probably triggered by exudates of the host plant [13]. On susceptible hosts, the fungus infects xylem parenchyma cells in a radial direction, from where generated hyphae reach and invade the xylem vessels [14]. *C. cacaofunesta* produces atypical smaller conidia which can pass through the cell wall pits and are probably involved in the rapid and massive plant colonization by the fungus [15]. As a *necrotrophic fungus*, *C. cacaofunesta* causes plant cell death during host colonization [2]. It can reproduce asexually, through vegetative propagation and conidia formation, and sexually [2, 16]. *Ceratocystis* species are homothallic due to an unidirectional mating-type switching mechanism resulting in the production of both self-sterile and self-fertile isolates [16, 17]. The ascospores are discharged by a long-necked perithecium and dispersed by insects from the genus *Ambrosia* [18, 19]. These insect vectors are attracted by volatiles compounds produced by the fungus [20].

Plant-fungal interaction studies have reported the formation of polysaccharide gels, tyloses, and phenolic compounds in the vascular vessels of plants infected by *Ceratocystis* species [15, 21]. CWC causes vascular obstruction that leads to wilting, vascular necrosis and tree death within a few weeks [2, 21]. However, in contrast to classical vascular fungi, such as *Verticillium spp.* and *Fusarium spp.,* which infect only the plant vascular system causing wilt disease, *Ceratocystis* species can colonize all stem tissues [14, 22, 23]. Proteins secreted by fungal pathogens have an active role in host tissue colonization and plant symptoms development.

Fungal secretomes are comprised of a diverse group of proteins involved in nutrient acquisition, self-protection, or manipulation of the biotic and abiotic factors [24]. Among the major classes of enzymes commonly found in fungal secretomes are carbohydrate-active enzymes, proteases, lipases, and oxidoreductases [24]. The specific composition of a fungal secretome is closely related to the microorganism lifestyle and its phylogenetic history [25]. For instance, through comparison of the genomes of three fungi that cause plant wilt disease (*V. dhaliae*, *V. albo-atrum* and *F. oxysporum*), Klosterman and coworkers (2011) identified a set of genes likely involved in niche adaptation. The identified genes enable pathogens to deal with osmolarity fluctuations and low content of nutrients in the xylem [23]. Also, *Verticillium* species secrete a large number of carbohydrate active enzymes with important roles in plant roots penetration, overcoming the plant defense responses and further colonization [23].

In pathogenic fungi, a wide variety of secreted proteins are considered pathogenicity and virulence factors because of their involvement in the disease development and modulation of the infection intensity, respectively [26]. Extracellular phospholipases are considered universal pathogenic factors in pathogenic fungi [27]. This denomination is due to their hydrolytic activity on membrane phospholipids of the host cell, causing its functional impairment or physical disruption, facilitating the invasion of the host tissues [28].

Phospholipases C (PLCs) hydrolyze glycerophospholipids at the phosphodiester bond, linking the glycerol backbone to the phosphate head group. The phosphate head group is also linked to a polar moiety [29]. Phosphatidylinositol (PI)-specific phospholipase C (PI-PLCs) are PLCs that cleave glycerophospholipids containing phosphoinositides as a polar head [29]. PI-PLCs have been found both in prokaryotes and eukaryotes [30]. However, enzymes from each group differ greatly regarding their structural properties, specific phosphoinositide substrates, released products and putative functional role. The fact that PI-PLCs from prokaryotes and eukaryotes are so different but carry the same name has caused great misunderstanding within the scientific community.

Enzymes from the bacterial PI-PLC family (EC 4.6.1.13) are calcium-independent and contain a single domain [29, 31]. These proteins cleave phosphatidylinositol (PI), lyso-PI and glycosyl-PI (GPI) lipids present in cell membranes. In pathogenic bacteria like *Staphylococcus aureus, Clostridium, Bacillus cereus* and *Listeria monocytogenes* PI-PLC are secreted proteins, being considered virulence factors [27, 30, 32]. On the other hand, eukaryotic PI-PLCs (EC 3.1.4.11) are proteins organized into several distinct domains, including PH, X, Y, and C2 [29, 30, 33]. In eukaryotes, these enzymes play a key role on cell metabolism through the regulation of cell proliferation and differentiation [33]. Eukaryotic PI-PLCs can only hydrolyze phosphorylated inositide PI 4, 5- bisphosphate (PIP2) releasing diacylglycerol (DAG) and inositol 1, 4, 5-triphosphate (IP3). DAG and IP3 are important secondary messengers required to trigger signal transduction pathways through the activation of protein kinase and intracellular calcium release, respectively [30, 33].

Moreover, most fungal genomes contain only a few genes coding for PI-PLCs and they have been associated with different functions: signal transduction, fungal development, pathogenicity and release of glycosyl-PI (GPI)-anchored surface proteins from target membranes [34–36]. For instance, the *Saccharomyces cerevisiae* genome contains a single PI-PLC gene (Plc1p) which encodes a protein with sequence and domain arrangement similar to the delta isoforms of mammalian PI-PLC [23]. Plc1p is involved in nutritional and stress-related responses [34]. The plant pathogen *Cryphonectria parasitica, which* causes chestnut blight disease, encodes *cplc1*, a multi-domain PI-PLC protein associated with mycelial growth and morphology [35]. The genome of another plant pathogen, *Magnaporthe oryzae,* codifies five PI-PLCs-encoding genes. The coded proteins are involved in signaling pathways with distinct roles in fungal development, conidiation and appressorium formation [36]. Higher numbers of secreted PI-PLCs have been identified in the genomes of *Fusarium oxysporum* (15 genes) and *Metharizium* species (8 genes), with possible involvement in fungal pathogenesis [22, 37]. With regards to protein organization, fungal PI-PLCs are still poorly studied.

In the present work, we analyzed the whole genome content of the cacao pathogen *C. cacaofunesta,* with particular emphasis on its secretome. Comparative analysis between the gene families of the *Ceratocystis* species and other Sordariomycetes shows a large expansion of extracellular PI-PLCs genes in the *Ceratocystis* genome and, remarkably, almost all PI-PLC genes are clustered in the same region of the genome. Our findings suggest that the evolution of pathogenicity in the genus *Ceratocystis* correlates with the expansion of the PI-PLC family. Additionally, we performed the prediction of structure and homology modeling of *Ceratocystis* PI-PLCs which suggested that this family of proteins must have phospholipids hydrolytic activity. Finally, we discuss possible roles of PI-PLCs proteins in the context of CWC. Altogether, our analyses provide new insights into the evolutionary and genetic mechanisms of *Ceratocystis* pathogenesis.

## Results

### Genome assembly and structure

*C. cacaofunesta* whole-genome shotgun sequences were generated using Illumina sequencing technology and assembled using Velvet [38]. The total length of the assembly was 30.5 Mb organized in 229 scaffolds larger than 1000 pb with N50 of 21 scaffolds (386 Kb size). The coverage of the genome was 369 fold: 166 fold sequencing coverage from 66.987.414 reads paired-end of 76 bp plus 203× fold coverage from 124.359.558 reads mate-pair of 50 bp. In total, 7382 gene models were predicted from the genome assembly. The overall *C. cacaofunesta* genomic statistics are summarized in Table 1.

**Table 1:** Genome descriptive statistics for *C. cacaofunesta*

Genome information	*Ceratocystis cacaofunesta*
Genome size Mb	30.5
Number of scaffolds	229
N50 Kb	386
% GC total	48.1
Number of gene models	7382
Genes with introns (%)	75.5
Assembly software	Velvet
Genome Coverage	369X

**Fig. 1:**
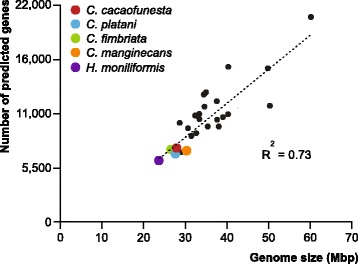
Correlation of genomes sizes and number of genes of *Ceratocystis* species compared with other Sordariomycetes genomes. List of species: CG, *Chaetomium globosum*; CM, *Cordyceps militaris*; CT, *Chaetomium thermophilum*; FG, *Fusarium graminearum*; FO, *Fusarium oxysporum*; FS, *Fusarium solani* (*Nectria haematococca*); FV, *Fusarium verticillioides*; GG, *Glomerella graminicola* (*Colletotrichum graminicola*); MC, *Metarhizium acridum*; MG, *Magnaporthe grisea*; MN, *Metarhizium anisopliae* (*Metarhizium robertsii*); NC, *Neurospora crassa*; NT, *Neurospora tetrasperma*; OP, *Ophiostoma piceae*; PA, *Podospora anserina*; TA, *Trichoderma atroviride*; TR, *Trichoderma reesei*; TT, *Thielavia terrestris*; TV, *Trichoderma virens*; VA, *Verticillium alfafae*; VD, *Verticillium dahliae* (Additional file 15)

The assembly size and number of predicted genes in *C. cacaofunesta* are similar to those of the previously sequenced *Ceratocystis* genomes (Fig. 1). As described, *Ceratocystis* species have smaller genome sizes and lower gene content when compared to other Sordariomycetes species. The genome size reduction in the *Ceratocystis* genus and in the closely related species *Huntiella moniliformis* might be directly associated to the lower gene content.

### Genome functional annotation

Gene models of *C. cacaofunesta* were annotated using AUTOFACT [39]. From the 7382 queries set, 6609 (89.5%) genes showed significant matches for gene descriptions (Additional file 1). Annotation of all the 6609 genes was performed using the Gene ontology (GO) and Kyoto Encyclopedia of Genes and Genomes (KEGG) databases to assign putative functions. From this total, 6007 genes were successfully annotated, yielding 3293 GO terms. The most abundant GO terms were related to organic cycling compound binding (1701), followed by heterocyclic compound binding (1699), ion binding (1471), and hydrolase activity (1031) (Additional file 2). Additionally, a total of 1411 genes were assigned to 115 KEGG metabolic pathways, and the following functions were highlighted based on the largest numbers of genes in these categories: biosynthesis of antibiotics (151), purine metabolism (128), pyrimidine metabolism (42), thiamine metabolism (41), oxidative phosphorylation (34), and pyruvate metabolism (32) (Additional file 2). This annotation was also performed for the type species of the *Ceratocystis* genus, *C. fimbriata*. The results show that the *C. fimbriata* proteome was distributed among GO and KEGG categories in a pattern similar to *C. cacaofunesta* (Additional file 2). Following sequence annotation, it was noted that Autofact pipeline could not assign a definition to about 10% of genes from both genomes (773 genes in *C. cacaofunesta* and 744 genes in *C. fimbriata*). These genes were assigned as “No-Hits” (Additional file 3). From the *C. cacaofunesta* set of No-Hits, transcripts were detected for 630 genes based on RNA-seq data of in vitro-grown mycelia, indicating that the respective gene models correspond to actual genes (Additional file 3).

The predicted proteomes for *C. cacaofunesta* and *C. fimbriata* were assigned to homolog gene groups using OrthoMCL. Gene groups suggest that these genomes share about 92% (6700) of their genes, from which 86% (6282 genes) had orthologs in *C. fimbriata*, and 6% (558 genes) had paralogs in the *C. fimbriata* genome. These paralog genes resulted from gene duplication after the divergence of these species (Additional file 4). Single-copy orthologs were distributed into 6282 gene families. Interestingly, we identified 112 and 25 exclusive genes for *C. cacaofunesta* and *C. fimbriata*, respectively (Additional file 4). Some of these genes, and also the unique genes of *C. cacaofunesta* (541) and *C. fimbriata* (600) that were not clustered in the Markov CLustering algorithm (MCL) groups, may be associated with the host relationship for each species.

**Fig. 2:**
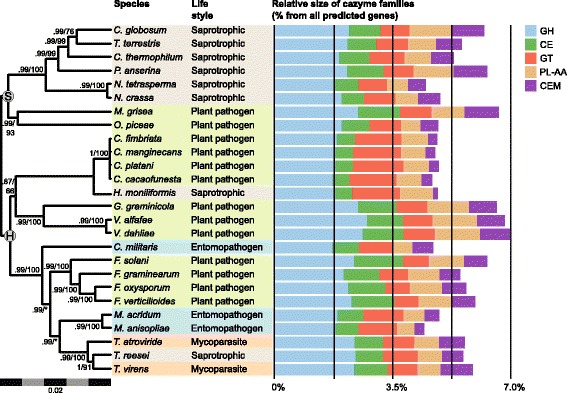
Amounts of CAZymes in each category defined using the dbCAM database. Sordariomycetes proteomes are classified as glycoside hydrolases (GHs), glycosyl transferases (GTs), carbohydrate esterases (CEs), carbohydrate-binding modules (CBMs), auxiliary activities (AAs), and polysaccharide lyases (PLs). The small bars in the right side of the figure are the fractions of CAZymes in each category relative to the total number of gene content in each species. The consensus phylogeny is in the left with branch supports obtained by bayesian posterior probabilities above branches and bootstrap support from likelihood analysis below branches. Nodes marked with S and H represents the taxonomic division in Sordariomycetidae and Hypocreomycetidae. In the center we present a table describing species life style, sapro, Saprotrophic; Entom, Entomopathogenic; P. path, Plant pathogen; Mycop, Mycoparasitism

Using the data base for automated carbohydrate-active enzyme annotation (dbCAN), we identified 275 predicted genes that encode potential carbohydrate-active enzymes (CAZymes) in the *C. cacaofunesta* genome, corresponding to approximately 3.72% of the predicted proteome of this fungus. Members of the *Ceratocysti*s genus and *H. moniliformis* have far fewer CAZymes than do any other *Sordariomycetes* analyzed so far. This reduction would be expected given the low gene content in the genome of these species. The calculated coefficient of determination between proteome size and CAZyme content for the *Ceratocystis* species was 0.89, suggesting that the observed decrease is mostly related to the smaller size of the predicted proteome. As expected, the number and distribution of the CAZymes families are very similar within *Ceratocystis* species (Fig. 2).

Next, we compared the CAZyme repertories of *Ceratocystis* species with their close relative, *H. moniliformis*, which has a saprophytic lifestyle. Interestingly, it was verified that, in general, *H. moniliformis* has fewer CAZymes (246) than the *Ceratocystis* species, which have 268 CAZymes on average. Also, the CAZymes repertories of these species are very different. For instance, the *Ceratocystis* species have a greater number of enzymes belonging to the CAZyme families AA4, CBM1, CBM20, GH18, GH16, GH32, GT8, CE13 and PL3, as compared to H. *moniliformis*. These CAZyme families are related to the conversion of phenolic compounds, cellulose-binding activity, granular starch-binding, chitinase, endo-1,3-β-glucanase, sucrose 1-fructosyltransferase, and pectinase functions, respectively. However, in CAZy families AA1, AA2, CE1, CE3, GH3, and GH43 - known as oxidases, esterases, β-glucosidases, and xylanases, respectively - the number of enzymes is smaller for *Ceratocystis* species (Additional file 5).

Cellulolytic activities were measured on cultures of *C. cacaofunesta*, *C. fimbriata* and compared to *Thielaviopsis paradoxa*. Our results showed that *C. cacaofunesta* and *C. fimbriata* display Carboxymethyl-cellulase (CMCase) and Filter Paper activities (FPase) when grown in avicel (cellulose crystalline containing significant fraction of amorphous cellulose) and xylan in 2:1 proportion (Additional file 5).

### Identification of genes potentially involved in plant-pathogen interaction

In order to identify genes with potential roles in pathogenicity in the *Ceratocystis* genome, three different approaches were applied: (i) given that key proteins involved in pathogenicity are usually secreted during the plant-pathogen interaction, the fungus secretome was predicted; (ii) the predicted secreted proteins were then validated using mass spectrometry, and (iii) a search for potential effectors among the predicted secreted proteins was conducted. Effectors were defined as small proteins that had fewer than 200 amino acids and high cysteine content (at least 4%).

**Fig. 3:**
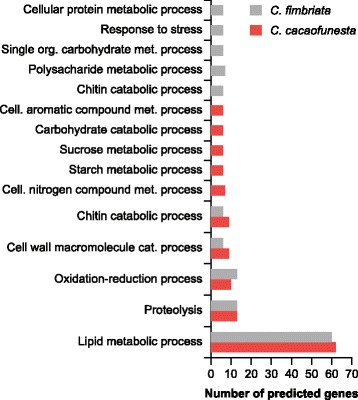
Overview of total secreted proteins in *C. cacaofunesta* compared with *C. fimbriata* classification using GO terms and Blast functional annotation results

The secretomes of *C. cacaofunesta* and *C. fimbriata* were predicted using SignalP and Target P v 1.1. The analysis identified 344 and 367 signal peptide-containing proteins in *C. cacaofunesta* and *C. fimbriata*, respectively. Thus, about 4.6% of the total proteome was predicted as being secreted for both microorganisms (Additional file 6). Gene ontology (GO) analyses showed that the most abundant GO term in the “Biological Process” category was related to lipid metabolic process (62 proteins). In the “Molecular Function” and “Cellular Process” categories, the most abundant GO terms were phosphoric diester hydrolase activity (56) and extracellular region (5), respectively (Additional file 6). Moreover, the proteins annotated with a GO functional term represented only 39% of the total predicted proteins in the secretomes; another 12.5% were no-hits, and about 36% of the predicted secretome was annotated as a hypothetical proteins. Figure 3 provides an overview of the *C. cacaofunesta* and *C. fimbriata* secretomes. In general, the distribution of GO term categories in the *C. fimbriata* secretome is similar to that of *C. cacaofunesta* (Additional file 6).

Mass spectrometry was used to validate the prediction of secreted proteins by *C. cacaofunesta*. The fungus was grown in a simulated xylem medium (SXM) and the supernatant was analyzed. The experiment identified 24% (86 proteins) of the total predicted secretome (342 proteins) (Additional file 6). This included 10 of the 43 No-hits predicted as secreted proteins.

By manual annotation, we identified several classes of proteins that were secreted by *C. cacaofunesta* and *C. fimbriata*. The most abundant class of proteins identified was the PI-PLC proteins. According to the conserved domain database (CDD) description, these proteins are related to the catalytic domain of the recognized virulence factors of pathogenic bacteria PI-PLC proteins. Interestingly, in bacteria, PI-PLCs are related to the host cell membrane degradation [40]. Four (4) of the *C. cacaofunesta* PI-PLCs were found in the extracts of cultures of the fungus grown in the inducer SXM medium. Also, we identified important proteins involved in the necrosis process: two proteins annotated as necrosis-inducing proteins (NPP1s) and one cerato-platanin protein (CP). Other proteins with potential roles in *Ceratocystis*-plant interactions are listed in Table 2. Similar results were obtained for *C. fimbriata*, including the high number of PI-PLC secreted proteins (Additional file 6).

**Table 2:** Potential secreted virulence factors in *C. cacaofunesta* and *C. fimbriata* genome

Virulence Proteins (Annotation)	*C. cacaofunesta* ^a^	*C. fimbriata* ^a^	Putative Role in Plants
Extracellular Serine-rich Protein	10 (1^b^)	23	Cell adhesion
GH 5,7, 11, 13, 16, 20, 28, 43, 53, 72, 61	25 (14^b^)	–	Cell wall degradation
GH 30,61,93,13,16,43	–	10	Cell wall degradation
Pectin Lyase	1^b^	3	Cell wall degradation
Ligninase H2	1	1	Cell wall degradation
Cellobiose Dehydrogenase	1^b^	nd	Cell wall degradation
PI-PLC	54 (4^b^)	53	Membrane degradation and hydrolysis of GPI-anchored proteins
Necrosis- and ethylene-inducing protein 2 precursor	2	2	Phytotoxic proteins
Ceratoplatanin	1^b^	1	Phytotoxic proteins/adhesion?
Glucan 1,3-beta-glucosidase	2	1	Polysaccharide gels degradation
Mixed-linked Glucanase	1	2	Polysaccharide gels degradation
Lacasse	1	–	Defense against oxidative Stress
Catalase	1	–	Defense against oxidative Stress
Multicopper Oxidase	1 (1*)	1	Defense against Oxidative Stress
AA4	2	nd	Degradation of phenolics compounds
Aspartic Protease	1	–	Proteolysis
Protease	4	–	Proteolysis (inactivated cutinases)
Alkaline Serine Protease	nd	1	Proteolysis
Potential Effectors^d^	85	nd	Suppress plant defense/Modulate plant physiology to fungal benefit

^a^Data from Secretome analysis, ^**b**^Identified by Mass-spectrometry; nd, not determined, ^d^ (Small proteins cysteine-rich)

Finally, following the aforementioned criteria, 85 proteins were identified as putative effectors in the *C. cacaofunesta* proteome. The functional annotation revealed that these proteins are related to: (i) glycoside hydrolase (GH) activities (alpha/beta-hydrolase, arabinan endo-1,5-alpha-L-arabinosidase, arabinogalactan, endo-1,4-beta-galactosidase, cellulose-growth-specific protein, covalently linked cell wall protein, endo-1,3(4)-beta-glucanase, endopolygalacturonase, proteins similar to mixed-linked glucanase, and a pectin lyase); (ii) proteins that could elicit plant responses (allergen Asp, cyanovirin-N domain protein, a protein similar to an expression library immunization antigen from *Colletotrichum gloeosporioides*, major allergen Asp, the Mmc protein); (iii) lipid metabolism (11 PI-PLCs, a phosphatidylinositol transfer protein, and a palmitoyl-protein thioesterase); (iv) proteins that could be involved in the resistance to plant responses such as oxidative stress (2og-fe oxygenase family protein, carbonic anhydrase, long chronological lifespan protein, Cu/Zn superoxide dismutase, proline oxidase, and short-chain dehydrogenase) and (v) proteases (Additional file 6).

### Comparative analyses reveal expansion of the PI-PLC family in *Ceratocystis* genomes

**Fig. 4:**
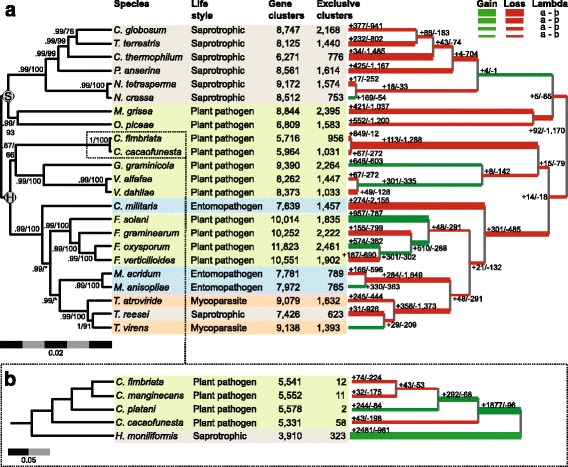
Phylogenetic inference and expansion/retraction of gene families for Sordariomycetes species. The consensus phylogeny is in the left with branch supports obtained by bayesian posterior probabilities above branches and bootstrap support from likelihood analysis below branches. Nodes marked with S and H represents the taxonomic division in Sordariomycetidae and Hypocreomycetidae. In the center we present a table describing species life style, with colored blocks separated according to monophyletic groups, and the number of protein family clusters and exclusive clusters obtained for each species. The phylogeny is mirrored in the right with branch width relative to the proportion of estimated gene gain (in green) and lost (in red). The absolute values estimated for protein families’ gain and lost are also indicated for each branch. **b** Phylogenetic inference and Expansion/retraction of gene family in *Ceratocystis genus*

Gene families that had potentially undergone significant expansion or contraction in the genomes of *C. cacaofunesta* and *C. fimbriata* relative to other Sordariomycetes were identified using the CAFE program (Computational Analysis of gene Family Evolution) [41]. The rates of gene gains and losses of several gene families, as well as the gene family size in the internal nodes of the phylogenetic tree using maximum parsimony, were estimated. A well-supported phylogenetic inference tree for Sordariomycetes was obtained (all internal branches had more than 90% bootstrap support) using both Bayesian and maximum likelihood analyses (Fig. 4a, left), positioning *C. cacaofunesta* and *C. fimbriata* species clustered with *Verticillium alfafae, Verticillium dahliae*, and *Glomerella graminicola*. Proteomes of all fungal species studied were grouped into 16,679 gene clusters shared by at least two species. The number of total gene clusters obtained and the exclusive clusters for each species are shown in Fig. 4a. The right part of Fig. 4a shows that the patterns of gain (lines in green) and loss (lines in red) of genes varied throughout the phylogeny, without an evident relationship to phylogenetic groups and species lifestyle. Most lineages showed more loss (thick red line) than gain (thin green line) of genes in the evolution process. The loss of genes was especially marked for the ancestral lineage of *Ceratocystis* in comparison to common Sordariomycetes ancestors (Fig. 4a, right). Approximately 4000 genes were lost in the *Ceratocystis* genus (Additional file 7). Figure 4b shows the phylogenetic inference for the *Ceratocystis* genus and *H. moniliformis*, which is described in the next section.

Gene families with differential evolutionary patterns for *Ceratocystis* species were further investigated. The top 10 protein families with significant expansion or retraction are listed in Table 3 (See Additional file 7).

**Table 3:** Major expanded and retracted gene families in C. cacaofunesta genome

Families	*Ceratocystis* size family	*Ceratocystis* ancestor size family	Difference	Family Annotation
Family_230	76	1	75	Phosphatidylinositol-specific phospholipase c
Family_5	100	41	59	Vegetative incompatibility protein
Family_86	58	4	54	FOT5 transposase
Family_187	33	2	31	GAG-POL Protein
Family_50	38	7	31	GAG-POL Protein – retrotransposon
Family_167	28	4	24	Serine/threonine-protein kinase Sgk2
Family_130	25	4	21	Protein Kinase
Family_45	26	10	16	Kinesin Light Chain - Acyl transferase/acyl lysophospholipase
Family_442	16	1	15	MULE transposase domain
Family_349	15	2	13	Acyl transferase/acyl hydrolase/lysophospholipase
Family_23	3	16	**−**13	Major facilitator superfamily
Family_12	13	27	**−**14	ABC transporter
Family_11	8	23	**−**15	Phosphoethanolamine n-methyltransferase
Family_10	3	19	**−**16	Heterokaryon incompatibility
Family_13	8	25	**−**17	MFS general substrate transporter – Multidrug transporter
Family_15	2	19	**−**17	Heterokaryon incompatibility
Family_17	3	23	**−**20	Beta-ketoacyl synthase
Family_9	8	29	**−**21	Major facilitator superfamily
Family_8	12	34	**−**22	Major facilitator superfamily
Family_6	9	33	**−**24	PTHR Dehydrogenase

The most expanded family is annotated as phospholipase-C, which corresponds to the most abundant protein class found in the predicted secretome: the PI-PLC protein family. The number of PI-PLCs increased significantly from two genes in Sordariomycetes to more than 44 copies in the *Ceratocystis* genus ancestor. Notably, within the most expanded families in the *Ceratocystis* genus, we found two other families of proteins with lysophospholipase activities that are potentially involved in phospholipids metabolism. Investigation of the enrichment of GO terms within the expanded families of *Ceratocystis* (Additional file 7) confirms that most enriched categories are related to the lipid metabolic process, phosphoric diester hydrolase activity, phosphoric ester hydrolase activity, and hydrolase acting on esters bonds. Other expanded families are related to phosphorylation; the metabolism of pantothenate; hydrolase activity; and the metabolism of vitamins. Within retracted families are protein families involved in transport and the oxidation/reduction process (Additional file 7).

### Comparative analyses with *Ceratocystis* and *H. moniliformis* genomes

**Fig. 5:**
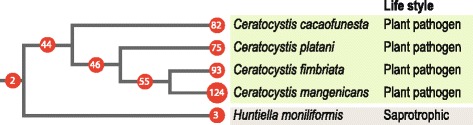
Evolution of PI-PLCs genes in *Ceratocystis* species and the close relative *H. moniliformis*. Numbers in the red dots displays the quantity of genes related to PI-PLC estimated using birth-death models by BadiRATE for each node

A second scale of comparative analysis was performed between species of the *Ceratocystis* genus and *H. moniliformis*. Predicted proteins of *C. cacaofunesta* were used as a training set to predict proteins in the previously published draft genomes of *C. fimbriata*, *C. platani*, and *C. manginecans* [42–44]. The predicted proteins of these species were compared to those of the related species *H. moniliformis* (Additional file 8). Their proteomes were grouped into 5077 gene clusters shared by at least two species. Among all clusters, 71% are shared by all species, and the other 29% have varied distributions among the *Ceratocystis* genus (Fig. 4b). Several gene families were significantly expanded and retracted within the *Ceratocystis* genus (Fig. 4b). A large number of genes that also appeared in the ancestor of the *H. moniliformis* lineage or in the ancestor of the *Ceratocystis* genus (3608 and 5077 gene clusters, respectively) was observed. Overall, *Ceratocystis* species showed greater similarity, sharing 3608 gene clusters (Additional file 8). Within the expanded families found in the *Ceratocystis* species, more than 300 genes belong to PI-PLC family, contrasting with the small number of these genes in the saprotrophic *H. moniliformis* (only three). In Fig. 5, the total number of PI-PLC genes observed for each species (red dots) and hypothesized for ancestors in the evolution process within the *Ceratocystis* genus is shown. The different numbers in each node reflect the fact that the expansion of this family occurred in the ancestor of the *Ceratocystis* genus and continued expanding into these 4 species.

### Characterization and genomic distribution of *Ceratocystis* PI-PLC genes

In the *C. cacaofunesta* genome, we identified 82 genes coding for proteins belonging to the PI-PLC family. The sizes of the PI-PLC proteins ranged from 157 to 487 amino acids, with an average of 350 a.a. From this total, 58 PI-PLCs were predicted to be extracellular proteins.

**Fig. 6:**
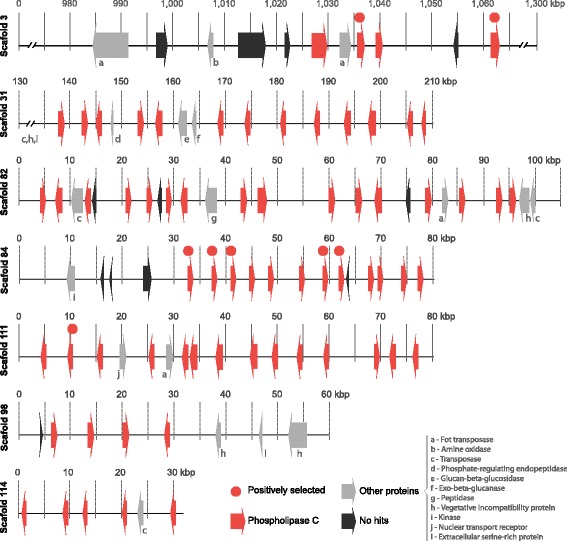
Schematic representation of major genomic clusters content containing PI-PLC genes in *C. cacaofunesta*. Numbers are in kilobases. These clusters involve 66 of the 83 PI-PLC genes (red arrows) and show a few other genes within (grey and black arrows)

All of the 82 PI-PLC genes of *C. cacaofunesta* were found to be distributed throughout 15 scaffolds. However, 79% of these genes (65) were found in clusters larger than 4 genes concentrated in just 6 assembled scaffolds (31, 82, 84, 98, 111, 114) ranging from 31 kb to 210 kb (Fig. 6). It was observed that the PI-PLC genes that are located close together within a scaffold encode proteins of similar sizes and have the same pattern of orientation. PI-PLCs clusters are composed mainly of PI-PLCs interleaved by large regions with no predicted genes but with many traces of broken transposases, identified from blast searches of these intergenic regions against the NR-NCBI database. Scaffolds containing clusters of PI-PLCs featured high densities of transposable elements (TEs), with the terminal inverted repeats (TIRs) being the most abundant class (Additional file 9). Analyses of TEs content and classification over *C. cacaofunesta* genome showed that TIRs were the third most abundant type of TEs (18%), following long terminal repeats (LTRs), retrotransposons (27%) and “unknown” type (25%). See Additional file 9 for complete TEs analysis.

About 40 other coding genes are distributed throughout these clusters (Fig. 6 and Additional file 10). Interestingly, the genes encoding proteins are related to noncellulolytic β-glucans hydrolysis (glucan 1,3-β-glucosidase and exocelular-β-1,3 glucanase); protein phosphorylation (kinase protein and serine/threonine kinase protein); or returned as “No-hits”. No difference in GC content was observed in PI-PLC-rich regions in comparison to the rest of the genome (Additional file 10).

All 6 scaffolds shown in Fig. 6 are highly similar due to the conserved domains of the PI-PLC genes and the presence of other tandem repeats, such as TIRs. It must therefore be considered the strong possibility that the genome assembler broke a single large genome region into different scaffolds because of the similarity and repeatability. This would mean that the PI-PLC gene clusters could be part of a single larger DNA fragment in *C. cacaofunesta* genome, with at least 560 kb. This region could actually be much longer, considering that other scaffolds contain a few PI-PLC genes. The same clustering pattern of PI-PLC genes was observed for other *Ceratocystis* species, but in many smaller scaffolds due to higher fragmentation of assemblies, suggesting that it could be an ancestral feature.

### PI-PLCs gene family evolution

**Fig.7:**
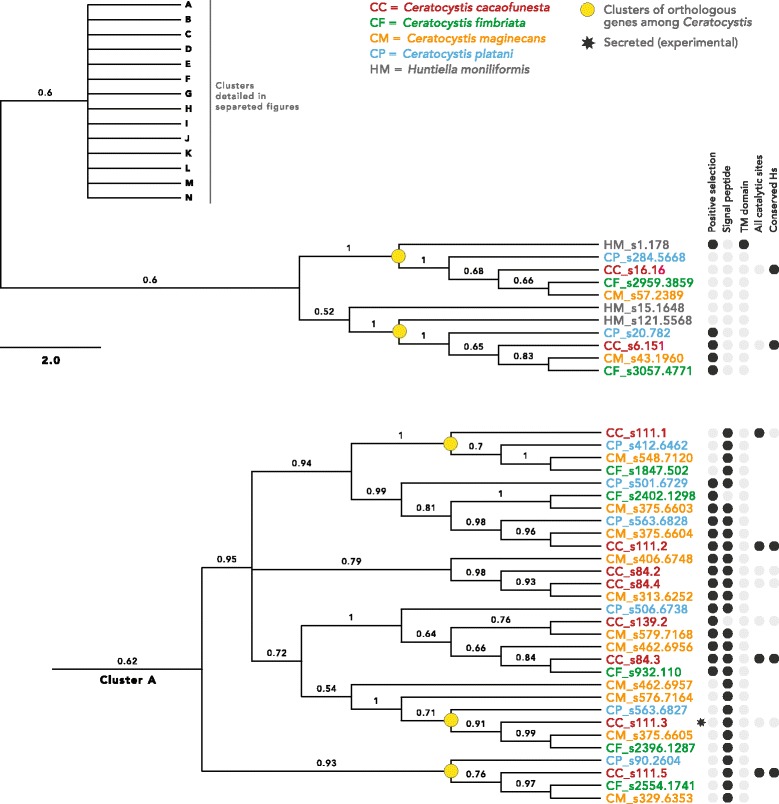
Phylogeny of PI-PLC gene family in *Ceratocystis* species. Fifteen clusters were defined being one ancestral, and the other 14 (A-N) equally related in a star-like branch. This figure shows the ancestral cluster and cluster A, being all other in Additional File 11. Posterior probabilities of the Bayesian Inference are above branches. A table in the right compiles information of the proteins

Because the expansion of PI-PLCs in *Ceratocystis* species occurred in the genus ancestor and continued in the species’ radiations and current lineages, we analyzed the evolution of this gene family in *C. cacaofunesta* and the other four members of the *Ceratocystis* genus. The phylogenetic hypothesis for *Ceratocystis* PI-PLCs predicts that at least 2 protein clusters are composed by ortholog genes in the *Ceratocystis* genus (Fig. 7). The phylogeny is characterized by one cluster of putative deep paralogs among all species that present a common ancestor before the divergence of *H. moniliformis* and *Ceratocystis*, and a related star-like tree formed by 14 clusters of proteins distributed along *Ceratocystis* species (Fig. 7; the complete phylogeny of the PI-PLC gene family is shown in Additional file 11). PI-PLCs genes of the *Ceratocystis* species showing a common ancestor with *H. moniliformis* are possibly the ones that retained ancestral characteristics. These ancestral-like PI-PLCs are composed of one group of orthologs among all *Ceratocystis* and *H. moniliformis*, and another group of orthologs among all *Ceratocystis* with a single duplication in *H. moniliformis*. There is no evidence for the presence of signal peptides in the ancestral-like PI-PLC and a *H. moniliformis* PI-PLC proteins (s2.351), although a transmembrane domain exists in one of them (Fig. 7).

The other 14 clusters of PI-PLCs are composed of proteins that have undergone duplications throughout the evolution of the *Ceratocystis* genus. The ortholog proteins among the four *Ceratocystis* studied are organized into 42 derivate clusters (yellow dots in Fig. 7) and are distributed among many clusters of paralogs that duplicate within one or more species. Paralogs closely related in the tree are positioned in the same scaffold and often next to each other within at scaffold. Evidence of signal peptides was found in many of the derivate PI-PLC clusters (Fig. 7).

Models of evolution by positive selection were tested against models of neutral evolution using *dN/dS* likelihood ratios in codeml. In this analysis, 58 PI-PLCs from the *Ceratocystis* genus showed significant values, indicating that they might have evolved under positive selection (Fig. 7; Additional file 11). Among the putative positively selected proteins, some are the ancestral-like PI-PLC with no signal peptide, and many others are recently duplicated proteins with signal peptide, indicating that these newer proteins may be secreted (Fig. 7; Additional file 11).

### Prediction of structure and homology modeling of *Ceratocystis* PI-PLCs

An integrated approach including gene annotation, homologue identification, and comparative modeling was employed to construct a putative and reliable structural model of the product encoded by the gene s111.3 from the expanded gene family of *C. cacaofunesta*. The PI-PLC encoded by the gene s111.3 was chosen for having been identified in both the predicted and experimental secretomes. The phosphatidylinositol-specific phospholipase C (PI-PLC, pfam ID PF00388.14) function of gene s111.3 product was predicted using the Web CD-Search Tool. The search indicated two well-conserved catalytic histidine residues. These histidine residues cleave the bond before the phosphate, converting phosphatidylinositol into inositol and a lipid in the phosphodiesterase reaction. These data strongly suggested that the product encoded by gene s111.3 is a phospholipase C protein. Hence, the structural model of putative phospholipase C protein from *C. cacaofunesta* was built using secondary structure optimized alignment.

A search for similar proteins was performed with the BLASTp algorithm. The search identified 31 proteins with available 3D structure related to phosphatidylinositol-specific phospholipase C. The template was selected using the following criteria: sequence identity, query cover quality, resolution of crystallographic structure, presence of ligands and protein class. According to these features, phosphatidylinositol-specific phospholipase-C from *Listeria monocytogenes* (Protein Data Bank ID: 1AOD) was selected as the best candidate.

**Fig. 8:**
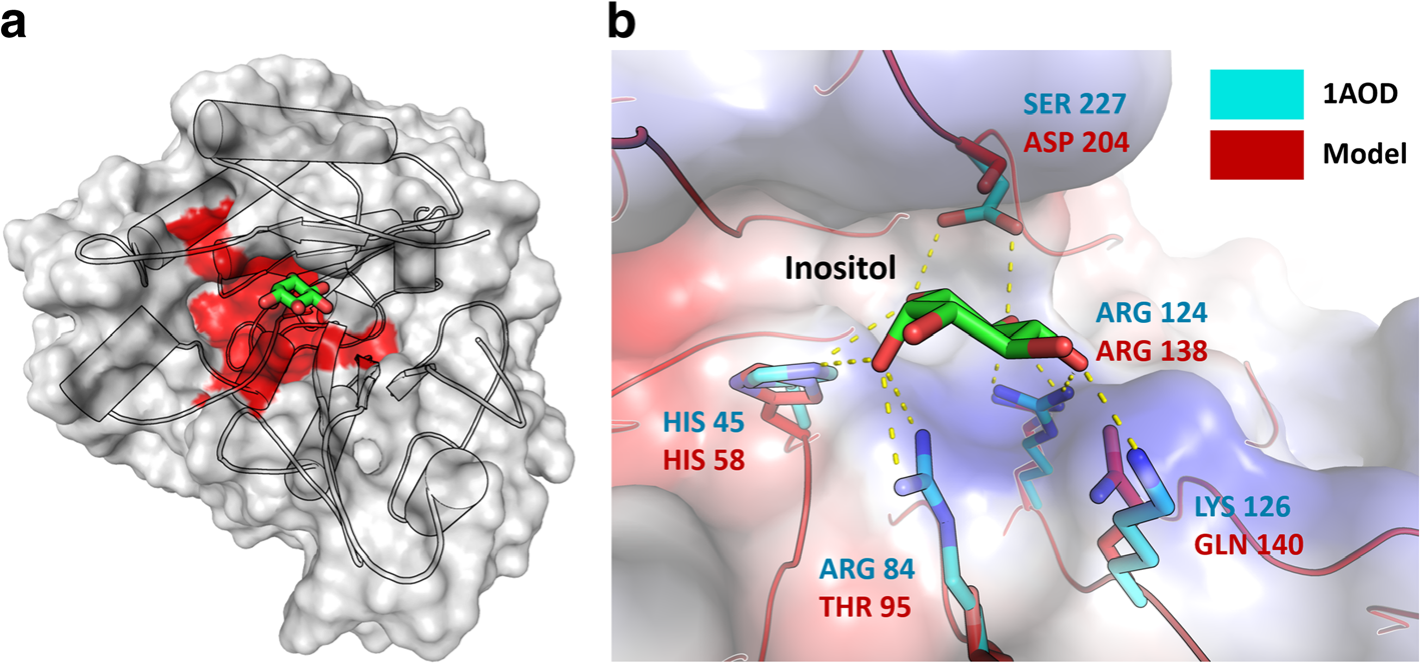
**a and b** *Ceratocystis* PI-PLC molecular model. **a**. (Left) Catalytic active region is shown in red interacted directly with the ligand inositol or phosphoinositol in Green. (Right) Detailed view of the active site of phospholipases C specific for phosphoinositol crystallographic (in cyan) and modeled (red). The detailed residues refer to the modeled protein. The mutations in Gln140 and Ser227 are conservative and capable of promoting interactions with the inositol molecule, necessary for the maintenance of phosphodiesterase activity. **b** Detailed view of the active site showed amino acid residues interact directly with the ligand. Amino acid residues of the template (Crystallographic resolve protein in PDB code 1AOD) are showed in cyan and PI-PLC *Ceratocystis* model in red. Mutations at Ser227 and Gln140 are conservative and able to promote interactions with inositol molecule, necessary for the maintenance of phosphodiesterase activity

The best model comprised 332 residues and showed an RMSD value of 1.4 Å over Cα for 369 equivalent residues of the *L. monocytogenes* putative homologue. The *C. cacaofunesta* PI-PLC model displays a typical TIM-barrel domain ((β/α)8-barrel) where the active site is located at the C-terminal end of the β-barrel. In *C. cacaofunesta* the enzyme forms a deep cleft that is lined mainly by polar and charged amino acid side-chains. The PI-PLC model from *C. cacaofunesta* indicated that the active site shares 85% sequence identity with the template. The active site included amino acid residues His58, Asn59, Thr78, Asp93, Thr95, Asn107, Arg138, Gln140, Asp199, Ser227, His257, Thr259 and Ser261 (Fig. 8a). Next, we employed molecular docking to verify whether the PI-PLC model from *C. cacaofunesta* would recognize inositol as substrate (Fig. 8b) [45]. The modeled binding modes indicate favorable polar interactions between inositol and active site residues (His58, Thr95, Arg138, Gln140 and Ser227) of the putative model. Moreover, the inositol binding mode to the PI-PLC model from *C. cacaofunesta* is very similar to the experimental binding mode observed in the binary complex of PI-PLC from *L. monocytogenes* [46]. Taken together, the presence of a predicted phosphoinositol binding domain, the conservation of catalytic site residues and the predicted ability to recognize inositol as substrate suggest that the protein encoded by gene s111.3 is a phosphoinositol specific phospholipase C [47, 48].

Finally, In order to gain some insight into the conservation of function within the expanded PI-PLC gene family, we compared the sequence of the gene s111.3 with the other 75 PI-PLC-encoding genes present in the *C. cacaofunesta* genome. The analysis generated a consensus sequence alignment which indicates the most common residues at a given position (Additional file 12). According to this alignment, the catalytic residues are well-conserved throughout the gene family, suggesting a common function. Subsequently, we used the CD-Search Tool Web server to search for conserved domains in the 75 genes [49, 50]. In all cases, the PI-PLC catalytic domain was found with a high degree of confidence [51]. Therefore, our data strongly suggest that the analyzed genes from *C. cacaofunesta* genome encode proteins that can bind phosphatidylinositol molecules.

## Discussion

*Ceratocystis* wilt infection is classified as an emergent disease due to the sudden increase in the number of afflicted plant species and geographical areas [52, 53]. Little is known about the biology and molecular mechanisms of this disease. Therefore, here we present the genome analysis of *C. cacaofunesta* with focus on the identification of pathogenicity genes which might facilitate plant colonization and provide resistance against plant defense responses. More specifically, we describe the expansion and evolution of the PI-PLC protein family in *C. cacaofunesta* genome, as well as in the other three *Ceratocystis* genomes published to date [42–44]. We provide evidence that the expansion of this gene family correlates with the evolution of pathogenicity in the *Ceratocystis* genus.

*C. cacaofunesta* genome*,* as well as the other *Ceratocystis* genomes published so far, has a small size and low gene content when compared to other filamentous fungi (Fig. 1) [42–44]. The genome size reduction is proportional to the decrease in gene content for all species studied. This is also true, for *Huntiella moniliformis,* a related species *that* also belongs to *Ceratocystidaceae* family. Evolution analysis of gene families showed that *Ceratocystis* has the highest number of extinct proteins among the compared genomes. In addition, the *Ceratocystis* species have similar genome features and seem to share similar genetic content. Genome functional annotation and orthology results revealed high homology within the proteomes of *C. cacaofunesta* and *C. fimbriata (*Additional file 4)*.* These similarities are in accordance with the short evolutionary distance among these species. A marked phenotypic difference between these species is that *C. cacaofunesta,* unlike *C. fimbriata,* has host specificity [1, 5]. Accordingly, slight differences between the proteomes of the two species were observed (Additional file 2) suggesting that this specialization might be due to some minor genetic variation. It has been described that in species with high genome similarity, host specificity might be related to variations in a single locus or in clusters of closely linked loci [54].

Interestingly, differences in TEs content between these species were observed. Our results show a five-fold expansion of TEs in the *C. cacaofunesta* genome compared to the *C. fimbriata* genome, suggesting that TE expansions may play a significant role in the structure, adaptation, and evolution of the *C. cacaofunesta* genome (Additional file 9). TE expansion may be involved in gene duplication, and gene loss inactivation [55, 56], all of which have worked to increase the genetic variability between the two species. Similar expansions have been reported in other Ascomycota fungal pathogens, such as *M. graminicola* [57], and they appear to accelerate the evolution of genes related to pathogenicity and host range [58, 59]. Genome functional annotation for these two *Ceratocystis* species revealed pathways related to thiamine and biotin metabolism (Additional file 2). These vitamins have been previously reported as being essential for the sexual reproduction of *C. fimbriata* [60]. Also, important pathways related to the biosynthesis of various terpenes and volatile compounds were found. The production of these compounds with fruity aromas has been reported for several *Ceratocystis* species [61].

In general, phytopathogenic fungi produce a variety of CAZymes during the plant colonization process. These enzymes are associated with plant cell wall degradation and perform crucial roles in the plant-pathogen interaction, being considered virulence factors [62]. Our results showed that the *Ceratocystis* species present the lowest numbers of CAZymes, when compared with other Sordariomycetes, including non-pathogenic species (Fig. 2). This difference was found to be related to the reduced gene content in the genomes of the *Ceratocystis* species, a trait that may be conserved within the genus. Nevertheless, the number of CAZymes observed for the pathogenic *Ceratocystis* species is still higher than that of their relative *H. moniliformis*, a saprotrophic species. Moreover, the composition of detected CAZyme families differs between the two genera. This would be expected given their different lifestyles. Many phytopathogenic fungi have even more CAZymes than do saprotrophic fungi, which are excellent degraders of plant biomass [63]. Furthermore, when compared to *H. moniliformis*, the *Ceratocystis* species have a higher number of CAZymes involved in the degradation of living plant tissues, such as pectinases. These enzymes are required for the degradation of pectin, a major component found between cells of living plant tissues [64]. *Ceratocystis* species have also a high number of genes classified in the AA4 CAZyme family, which contains vanillyl-alcohol oxidases that catalyze the conversion of a wide range of phenolic compounds. Histological studies involving *Ceratocystis* infection showed the production of these compounds via the plant mechanism of defense against *Ceratocystis* pathogens [21].

Another interesting fact discovered in the analyses of CAZymes is that there is a large difference when comparing the *Ceratocystis* species with the classical or true vascular wilt pathogens (TVPs) of *Verticillium* and *Fusarium.* Traditionally, the *Ceratocystis* species have been classified as vascular wilt pathogens due to the wilting disease they cause through the impairment of xylem vessels. However, recent reports showed differences related to the infected tissue; while TVPs infect xylem vessels, *Ceratocystis* infect xylem parenchyma [21, 22]. Also, some *Ceratocystis* species are able to infest other plant organs, suggesting that they are not true vascular pathogens (NTVPs) [14, 65]. This difference implies divergent pathogenesis strategies among TVPs and NTVPs. Ours analysis presents some molecular evidence that supports these differences. Klosterman and coworkers [23] identified the expansion of polysaccharide lyase (PL) in *Verticillium* species, which have an enhanced capacity to degrade plant pectins. Meanwhile, *Ceratocystis* have fewer pectin lyases and do not present PL11 homologs, which according to the authors were present only in the wilt pathogens [23]. Additionally, *C. cacaofunesta* (and *C. fimbriata*) predicted proteome does not contain homologous to the specific wilt proteins involved in the maintenance of osmotic stability and the adaptation of TVP to their ecological-niche [23] (Additional file 13).

Otherwise, important virulence factors were found in the secretomes of *C. cacaofunesta* and *C. fimbriata* (Table 2), including a unique cerato-platanin (CP), a known phytotoxic protein. CP induces necrosis in tobacco leaves [66] and it has also been proposed that it may allow the attachment of hyphae to hydrophobic surfaces during the formation of aerial structures [67]. Interestingly, *Ceratocystis* species do not have genes encoding class I and II hydrophobins suggesting that other proteins, such as CP, could be involved in cellular adhesion.

Additionally, two genes encoding proteins similar to NEP1-like proteins (NLPs) were identified. NLPs are classified as Type 1NLP when contain two cysteine residues in the primary sequence and as Type 2NLP when there are four cysteine-residues [68]. We identified that *C. cacaofunesta* and *C. fimbriata* have a copy of each type of NLP. The first identified NLP (NEP1) was isolated from a *F. oxysporum* culture filtrate. This protein was capable of producing necrosis and ethylene induction in the leaves of dicotyledonous plants [69]. Nowadays, NLPs are accepted as cytotoxic proteins, even though their mechanism of action is not well understood [70].

Among the CAZyme families detected in the secretome of *C. cacaofunesta*, GH5, GH11, GH16, GH43 and GH61 are related to deconstruction of cellulose and hemicelluloses (Additional file 5 and Additional file 6). Thus, we measured the cellulolytic activity on cultures of *C. cacaofunesta* and *C. fimbriata* and compared it to that of *T. paradoxa*, a sugarcane pathogen known as a cellulase producer [71]. Our results showed that *C. cacaofunesta* and *C. fimbriata* have cellulase activities when grown in conditions which reflect the cellulose:hemicellulose composition of the plant cell wall (Additional file 5). We also identified a single ligninase, suggesting that lignolytic activity is limited in these fungi, as described for *Ophiostoma* species [72].

Other important virulence factors required for plant colonization by fungi are effectors. An effector is defined as any secreted molecule that modulates the interaction between the pathogen and its host [73]. The *C. cacaofunesta* genome has a great variety of proteins with effector-like characteristics. Within this category we found proteins such as the allergen Asp and cyanovirin, which can elicit plant responses, and also proteins possibly involved in resistance to host-generated oxidative stress. Interestingly, many proteins with effector-like characteristics found in *C. cacaofunesta* did not match published sequences suggesting that these might be interesting targets for further studies.

The phylogenetic relationship within the *Ceratocystis* genus was also studied. The obtained results showed that this genus diverged very recently, when compared to other Sordariomycetes genera, such as *Fusarium* and *Trichoderma* (Fig. 4a). The relationships we observed are in agreement with de Beer (2014) phylogeny. The Bayesian phylogram of the mini-chromosome maintenance complex component 7 (MCM7) dataset revealed *C. cacaofunesta* as more divergent to *C. fimbriata* than *C. manginecans*. Besides their phylogenetic proximity, *C. manginecans* and *C. fimbriata* were described as different species [5]. However, in recent populational and phylogenetic investigations of the Latin American Clade of *Ceratocystis*, *C. platani* and *C. cacaofunesta* were proposed as individual species but *C. manginecans* was considered a lineage within *C. fimbriata* complex, implying that *C. fimbriata* would be a host generalist pathogen [74, 75]. As for many others pathogen where host specificity drives genetic drift and individualisation of species, such species delimitations are difficult issues.

Despite the short evolutionary time for divergence, significant genomic structural changes occurred in the *Ceratocystis* ancestral lineage. First, the genome of the *Ceratocystis* and *Huntiella* genera underwent a large reduction in size and gene number. A large proportion of genes was lost across gene families, many of which were involved in transport, detoxification and oxidation/reduction processes. Curiously, these processes are considered important for fungal plant pathogens, as they may play a crucial role in counteracting the oxidative stress generated by the host plant [58]. Despite the observed reduction in gene content, some gene families have shown a remarkable expansion in the *Ceratocystis* genome. This is especially true for the PI-PLC gene family (Fig. 5). Further analysis into the expansion and retraction of gene families showed that both the *Ceratocystis* and the *H. moniliformis* ancestors presented a large number of new genes. However, only the *Ceratocystis* ancestor displayed significant expansion of the PI-PLC gene family. The initial duplication of a PI-PLC enzyme, potentially surrounded by transposons, began a process of extensive transpositions in the genome, increasing the PI-PLC copy number in a short period of time. This expansion continued in the resulting *Ceratocystis* lineages (Fig. 5). All the transposition events and PI-PLC duplications created genomic clusters that might actually be connected in a large single fragment or even a chromosome. This large sequence was divided into different scaffolds by assembly issues with repetitive regions (Fig. 6).

The rapid expansion of the PI-PLC gene family is also indicated by the star-like phylogenetic relationships among 14 PI-PLC clades, contrasting with the well-defined branching pattern from those of *Huntiella* and those that diverged within the 14 clades. Many PI-PLC genes that are closely related in gene phylogeny are also positioned near each other in genomic clusters, suggesting an evolution by duplications in tandem (Fig. 7). Pathogenic filamentous fungi present effector proteins that evolved on genomic regions that are not required for saprotrophic growth. These regions are called conditionally dispensable chromosomes (CDCs), in contrast to chromosomes whose gene content is essential and conserved across species [22]. CDCs have been associated to pathogenicity and host-range delineation in *Leptosphaeria, Alternaria*, and *Fusarium* species [76–78]. We are now working on obtaining the chromosomal map of *C. cacaofunesta* in order to analyze the possibility of the PI-PLC genes being located on CDCs.

Expansion of secreted PLC proteins is not a rare event in the genomes of fungal plant pathogens. For instances, the wilt pathogen *Verticillium dahliae* carries 19 genes encoding Patatin-like phospholipases which are likely involved in pathogen growth, lipid metabolism and signaling. Interestingly, 15 of these genes are located in *V. dahliae* lineage-specific regions (LS), which are enriched in repetitive sequences [23]. This PLC family expansion could be related to pathogenic properties required for the development of *Verticillium* wilt disease [23]. *Fusarium oxysporum* also exhibits expansion of several protein families related to lipid metabolism [22]. Specifically, eight PI-PLC encoding genes were described, all of which are located in LS regions [22]. Moreover, the entomopathogenic fungus *Metarhizium anisopliae* presents 12 genes encoding secreted lipases, some of which are similar to PI-PLC and could be involve in pathogenicity [37]. *Ceratocystis* species have a remarkable number of secreted PI-PLC genes, well above the average found in other fungi. Also, unlike the proteins from other fungi, *Ceratocystis* PI-PLCs show a significant similarity to the protein domain of pathogenic bacteria, which are known virulence factors.

The extreme expansion in PI-PLC gene number and divergence in gene sequence, both in the ancestral genus of *Ceratocystis* and in current species, point toward a diversification process that might be adaptive for pathogen host infection. Considering that *Huntiella* species are not pathogenic, the genomic revolutions that expanded PI-PLC genes may have functioned as a pre-adaptation to the *Ceratocystis* lineages that evolved as necrotrophic pathogens. We obtained structural evidence that all of these newly duplicated proteins could be functional and that positive selection might be driving their evolution, with the accumulation of various non-synonymous substitutions in their sequences (Fig. 7). Pathogenicity-related proteins are expected to show high rates of non-synonymous substitutions. These changes are associated with the process of positive selection that drives the arms race established between the pathogen and its host [79]. Evidence of positive selection has been widely reported for plant pathogens in virulence genes, especially for effectors [80].

Because the new non-synonymous substitutions diverge greatly among *Ceratocystis* that infect different hosts, we infer that different selective pressures may be acting on each lineage, possibly as a result of specific interactions between the pathogen and its host. Considering that *C. manginecans* might be part of *C. fimbriata* species complex [74, 75], differences in PI-PLC numbers and evidences of selection would not be a species-specific feature but a lineage specific one.

PI-PLC proteins found in *Ceratocystis* species have structural features and domain organization similar to those of their homologue (LmPI-PLC) from the prokaryotic *L. monocytogenes* (Fig. 8a, b). According to the obtained structural model, genes from the *C. cacaofunesta* PI-PLC family are likely able to bind phosphatidylinositol molecules. Our data indicates that these PI-PLC proteins have all the structural features to perform typical functions of bacterial PI-PLC: (i) catalyze the cleavage of phosphatidylinositol (PI) (or its phosphorylated derivatives) to produce DAG and the water-soluble head group phosphorylated myo-inositol [31]; and (ii) catalyze the release of proteins tethered to membranes by GPI-anchor proteins [81]. It is important to emphasize that PI-PLC acts on a substrate that does not occur in solution, but is rather found in an aggregated state, as they are present in the cell membranes [82]. Therefore, PI-PLC from *C. cacaofunesta* might be able to hydrolyze phospholipids in cell membranes, leading to their disruption or dysfunction, similarly to their bacterial homologues. Moreover, functional analysis showed that LmPI-PLC, unlike classical bacterial PI-PLCs, has a very low hydrolysis activity on GPI-anchored proteins [83]. This protein feature was associated to the lack of a small beta-strand (Vb), which it is presents in all bacterial PI-PLC [83]. Our analysis showed that *Ceratocystis* PI-PLCs contain the 8 amino acids region capable of forming the small beta-strand (Additional file 12). Therefore, *Ceratocystis* PI-PLCs might also be able to release proteins anchored to membranes by glycosylphosphatidylinositol (GPI).

GPI-anchored proteins are ubiquitous and include enzymes, receptors, differentiation antigens and other biologically active proteins [84]. In fungi like *Penicillium roqueforti*, *Paecilomyces variotii* and *Aspergillus niger,* it has been found that GPI-anchored proteins are processed at the plasma membrane by a phosphatidylinositol-specific phospholipase C [85]. Many fungal cell wall proteins are covalently bound to β-1,6-glucan via a remnant of a GPI anchor. These proteins are involved in important cellular processes, such as adhesion, invasion, biofilm formation, and flocculation [86–92]. In *C. cacaofunesta*, a total of 97 putative GPI-anchored proteins were identified. These were found to have similarly with various proteins groups, including chitin synthases, GH16, GH72, pepsin-like proteinases, Alpha-L-arabinofuranosidase, permeases and cysteine rich proteins (Additional file 14). Therefore, *C. cacaofunesta* GPI-anchored proteins might be involved in fungal morphogenesis and invasion. On the other hand, GPI-anchored proteins in plants are involved in many processes [93]. For instance, *Arabidopsis thaliana* GPI-anchored proteins are related to cell wall deposition and remodeling, defense responses, and cell signaling [93].

**Fig. 9:**
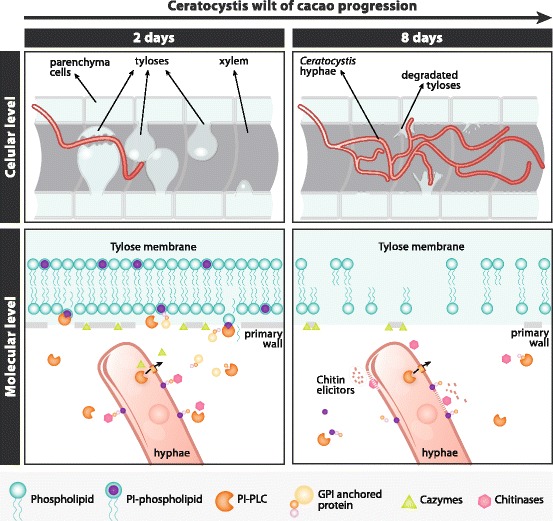
Hypothetic model for a possible role of secreted *Ceratocystis* PI-PLCs in the context of *Ceratocystis* Wilt of cacao. The fungal moving into parenchyma of vascular system grow and degrading life primary host plant walls, using a variety of CAZymes including celullases. PI-PLCs proteins have the putative ability to recognize inositol and catalyze the cleavage of phosphatidylinositol (PI) substrates present in the host membranes and could rapid destroy the stability of the cell, specifically the typical structured berried formed by the plant called tyloses. The genome arsenal together with several secreted PI-PLCs proteins produced by *C. cacaofunesta*, rapidly causing necrosis and degrading the vascular system in cacao plants. Also *C. cacaofunesta* PI-PLCs potentially cleave glycosylphosphatidylinositol (GPI) anchors proteins present in the surface of the both fungal and plant membranes. These GPI released proteins could for instance produce chitin residues which could elicit plant defense responses, GHs could help to hydrolyze the plant cell wall an arsenal of proteins involved in plant-fungal interaction. Ultimately, secreted PI-PLC of *C. cacaofunesta* could amplify both signals of the pathogenesis of the fungus and host defenses

Based on the data obtained throughout this work and the available information of the histopathology of the *C. cacaofunesta* - cacao interaction, we suggest a model that would explain the possible roles of the PI-PLC family of proteins in the wilt disease context (Fig. 9). A histopathological study of the interaction between *C. cacaofunesta* and cacao showed plant responses to *C. cacaofunesta* infection in both resistant and susceptible plants [15]. Plant responses included the discoloration of the primary walls of infected xylem vessels and the surrounding parenchyma cells; the mobilization of polyphenols in parenchyma cells, the translocation and accumulation of starch in the xylem and the production of gums and tyloses [15]. The main differences between genotypes were the intensities and kinetics of the appearance of those responses being more pronounced in the susceptible varieties [15]. In susceptible plants, the mycelium penetrated cells that were adjacent to the xylem and reached the xylem and parenchyma cells, overcoming structural resistance barriers such as tyloses. Tyloses are saclike structures that develop when turgor pressure causes parenchyma cell outgrowths through vessel parenchyma pit pairs into the lumen of xylem vessels [94]. During tyloses formation, the primary wall component of the pit membrane is not pushed into the vessel. Instead, a fine protective layer containing pecto-cellulosic material is deposited between the protoplast and the pit membrane [94]. With regards to tyloses formation and *Ceratocystis* wilt of cacao, Santos and coworkers (2013) observed that in susceptible plants, vessels containing the fungus were almost clear of tyloses. Nevertheless, adjacent vessels were completely occluded with tyloses formed in a matrix of gum 7 days after the inoculation [15]. Here, we hypothesize that PI-PLC could be involved in cellular disruption and tyloses degradation aided by secreted proteins that degrade the cell wall. Also, PI-PLC might release proteins anchored to GPI, both in the fungal and in the plant cell walls, enhancing the process of tissue degradation and allowing the fungus to advance to regions more distant from the point of inoculation (Fig. 9). Collin & Parke (2008) suggested that *Phytophthora ramorum* produces enzymes able to degrade tyloses [95]. *C. cacaofunesta* PI-PLCs would contribute to amplify both, the signals produced by the pathogen and the host defense responses. We are currently gathering experimental data to support this model. In addition, necrosis in parenchyma cells may be assisted by other proteins reported in this work, such as different NEPs, cerato-platanin and the various types of CAZymes, which might assist in the degradation of the primary cell wall. All of the PI-PLCs hypothesized functions would contribute, together with the other secreted enzymes, to the development of wilt symptoms and the subsequent death of the host plant.

In this scenario, it must also be considered the significance of reaction products released from the hydrolysis of the membrane phospholipids by PI-PLCs during plant-fungus interaction. Myo-inositol and its derivatives have important roles in eukaryotic cells [96]. For instance, these compounds impact plant growth and development [97], sexual reproduction in some fungi like *C. neoformans* [98], and fungal cellular function and pathogenicity [99]. These examples suggest that the impact of PI-PLCs proteins on the development of *Ceratocystis* wilt of cacao is probably much greater than it is hypothesized here. Future investigations are aimed at elucidating experimentally the pathogenicity mechanisms related to PI-PLC.

The diverse arsenal of pathogenic genes present in *C. cacaofunesta* and *C. fimbriata* genomes supports the idea that CWC is produced by the synergistic effect of toxin activity with parenchyma degradation and their fast growth and sporulation, with the consequent destruction of the plants vascular system, as reported in histopathological studies [15]. All of these mechanisms might contribute to make of *Ceratocystis* species lethal pathogens.

## Conclusions

Here, we presented the *C. cacaofunesta* whole-genome analysis. To our knowledge, this is the first work to report (i) the expansion of PI-PLC genes in *C. cacaofunesta* and other *Ceratocystis* species; (ii) PI-PLC genes distribution in *Ceratocystis* genome forming clusters in regions rich in TEs; (iii) their similarities with PI-PLC pathogenicity factors from bacteria; (iv) the evolutionary analysis of PI-PLC in Sordariomycetes suggesting that PI-PLCs in *Ceratocystis* are involved in pathogenicity; (v) the relationship between evolution of pathogenicity in this genus and the PI-PLC gene family expansion; (vi) molecular model of a fungal PI-PLC, from *C. cacaofunesta*, suggesting that they could be functional proteins, possibly involved in membrane dysfunction and release of GPI-anchored proteins like their bacterial homologues. Based on our data and histopathological information about *C. cacaofunesta-cacao* interaction, we proposed a model suggesting possible roles for the PI-PLC protein family in CWC. The ultimate result of their action would be the amplification of both pathogenic signal and host response*.* Still, the validation of the proposed mechanisms demands additional experimental studies. Lastly, we also identified other potential virulence factors in the *C. cacaofunesta* genome. Altogether, our work contributes to the understanding of the mechanisms underlying phytopathogenicity in tropical crops, providing new perspectives for interference with plant-pathogen interactions.

## Methods

### Strain and nucleic acid isolation

The strain C1593 of *Ceratocystis cacaofunesta* used in this study was isolated from infected cacao trees of a cultivation located in the district of Uruçuca, Bahia, Brazil. The strain was generously donated by Dr. Tomas Harrington, from the Iowa State University Department of Plant Pathology. The methodology for nucleic acid isolation was previously described by Ambrosio and coworkers, 2013 [100].

### Genome sequencing and sequence assembly

The genomic DNA was sequenced on a Genome Analyzer IIx platform (Illumina) at the University of North Carolina, Chapel Hill High-Throughput Sequencing Facility. A whole-genome shotgun strategy was used to produce 76-bp paired-end reads (400-bp insert size) and 50-bp mate-pair reads (3-kb insert size). The paired-end reads were assembled into longer contigs using Velvet version 1.0.12 [38], with an optimized k-mer size of 65. The pre-assemblies were used to construct scaffolds with the SSPACE program [101].

### Gene prediction and annotation

The *C. cacaofunesta* genes were predicted by combining evidence retrieved from RNA-seq data and comparative gene finding. RNA-seq reads were obtained via in vitro laboratory experiments. RNA isolation, and RNAseq generation protocols were previously described by Ambrosio and coworkers (2013) [100]. A second set of RNAseq data was generated in a parallel project for which a manuscript is in preparation. These data were used in this work to improve gene prediction. Mapping these reads into the assembled genome using STAR [102] produced splicing junction information, which were used in the self-training step of Genemark-ES [103] for the first prediction round. We used the BRAKER1 pipeline [104] with the splicing junctions’ information and the predictions from Genemark-ES to create a list of reliable genes. Next, we selected 1300 genes with Blastp [105] alignments (e-value cutoff of 1e^− 5^) to proteins from close relative species obtained from a non-redundant (nr) database (NCBI), filtered by those with end-to-end alignment, gaps smaller than 15 bases, and at least 10 hits for each query. From the final list, 1000 genes were used as the training set in the gene predictor Augustus 3.1 [106], and the remaining 300 genes were used as the test set. We checked for the distribution of gene sizes in the training set to match the gene size distribution obtained from the first-round prediction of Genemark-ES. The predictions from Genemark and Augustus and also information from RNA-seq were combined by EVidenceModeler (EVM) [107], resulting in the final prediction. For the other *Ceratocystis* species we analyzed, predictions were done with Augustus 3.1 using the training list of genes from *C. cacaofunesta*.

The Autofact program [39] was used to perform automatic annotation of the *C. cacaofunesta* gene prediction. The main contribution of Autofact is its capacity to resume the annotation based on sequence similarity searches in several databases. For this, we used Blastp (e-value cut-off of 1e-5) to align the genes against protein databases, including the NR/NCBI, KEGG [108], UniRef90 [109], and CDD/Pfam [110].

### Protein general functional analyses

Protein classification of the *C. cacaofunesta* genome by gene ontology and the KEGG metabolic pathway was carried out using BLAST2GO tools [111].

### Carbohydrate enzyme CAZy-families analysis

CAZymes content was predicted in 26 Sordariomycetes proteomes. Lists of all species and Data Bank access number of genome sequence are available in Additional file 15. The CAZyme analyses were performed using the HMMER3 and dbCAN HMMs databases, which are available online at the dbCAN homepage [112]. The amounts of CAZy enzymes in each CAZy category for each species were obtained from the dbCAN output.

### Predicted secretome

Secreted proteins were identified by the prediction signal peptide/non-signal peptide using Signal P Version 4.1 [113]. The automatic annotation of the secretome was performed using the NR/NCBI, KEGG [108], and UniRef90 databases (BLASTp with an e-value cutoff of 1e-5) [114] and summarized using the AutoFACT program [39]. The CDD/Pfam database was used to identify the conserved domains. The Blast2GO program [111] was used to classify the gene ontology.

### Secretome experimental identification using LC-MS/MS

Was identified the secreted proteins produced by *C. cacaofunesta* in media supplemented with cacao plant extracts. First, 1 g of mycelia fungus was grown in flasks containing 100 mL of simulated xylem medium [115] containing 2 g/L sodium polypectate, 4 g/L vitamin-free casein hydrolysate, 1× potassium salt, 1× MPR trace minerals, 1 ml/L, of Vitamins 100× solution, 1 ml/50 ml amino acid standard solutions, and 0.1 μM biotin for 7 days at 28 °C. The mycelia were collected, washed with sterile water, inoculated into new aliquots of the same media supplemented with 1% (*w*/*v*) final concentration of *Theobroma cacao* xylem extract with a sole carbon source, and collected. The xylem extracts were obtained by maceration of sterile cacao stem from 5-month–old cocoa seedlings. After a 72-h period of incubation into the inductor media, *C. cacaofunesta* mycelia were separated from the liquid media by filtration through a paper filter (*Whatman* no. 1) and centrifuged for 15 min at 2000 g.

The supernatants were concentrated 100-fold using (Vivaspin Concentration- GE) and quantified by Bradford assay. 295 μL of protein from each sample was denatured, as described previously [116]. The resulting peptide solution was dried in a SpeedVac concentrator and resuspended in 100 μL of 0.1% formic acid. An aliquot of 4.5 μL was separated using C18 (75 μm × 100 mm) RP-nanoUPLC (nanoACQUITY, Waters) coupled with a Q-Tof Ultima mass spectrometer (Waters) with a nano-electrospray source. The flow rate was 600 nL/min, and the gradient was 2–90% acetonitrile in 0.1% formic acid over 45 min. The instrument was operated in the “top three” mode, in which one MS spectrum was acquired, followed by an MS/MS analysis of the three most intense peaks [117].

The spectra were acquired using MassLynx v.4.1 software, and the raw data files were converted to a peak list format (mgf) using the Mascot Distiller v.2.3.2.0, 2009 software (Matrix Science Ltd.) without summing the scans, allowing for a label-free analysis. The files were then searched against the *C. cacaofunesta* database (7321 entries, 7269 proteins) using Mascot engine v.2.3.01 (Matrix Science Ltd.). Carbamidomethylation was used as a fixed modification, along with methionine oxidation as a variable modification, one missed trypsin cleavage, and a tolerance of 0.1 Da for both precursor and fragment ions. For the protein quantitation, the .dat files from the Mascot output were analyzed using Scaffold Q+ (version 3_00_03, Proteome Software), and quantitative values (normalized spectral counts) were obtained [118, 119]. For the endogenous peptide identification, methionine oxidation was set as a variable modification, with a tolerance of 0.1 Da for both the precursor and fragment ions. Only peptides with a minimum of 5 amino acid residues and statistical significance (*p* < 0.05) based on the Mascot scores were considered in the results.

### Identification of potential effectors

Potential effectors were sought using the following parameters: small secreted proteins with cysteine-rich residues that are less than 200 amino acids in length and contain at least 4% cysteine residues, according to Klosterman et al. (2011) [23]. Additionally, GPI (glycosylphosphatidylinositol)-anchored proteins were identified by the GPI-anchor attachment signal among the predicted secreted proteins using http://gpcr.biocomp.unibo.it/predgpi/ [120].

### Comparative phylogenomics

Comparative analysis were carried out at two different scales: at a large scale, comparing *C. cacaofunesta* and *C. fimbriata* to 21 other species from the Sordariomycetes class to identify *Ceratocystis* novelties; and at a small scale, comparing *C. cacaofunesta* to four other species from the *Ceratocystis genus* to understand the evolution of novelties within the genus. List of all species and Data Bank access number of genome sequences are available in Additional file 15.

### Genome sequences

In the large-scale analysis, we used the genome sequences of 23 Sordariomycetes *species* available from the JGI (Joint Genome Institute) database. Accessions codes and references are available in Additional file 15. In the small-scale analysis, we used the genome sequences of 3 *Ceratocystis* species (Table 1)—*C. manginecans* [GenBank: JJRZ01000000]; *C. fimbriata* [GenBank: APWK00000000], and *C. platani* [GenBank: LBBL01000012.1]—which are available from the GenBank database of the National Center for Biotechnology Information (NCBI) (http://www.ncbi.nlm.nih.gov). The *H. moniliformis* data are available in the accession number JMSH00000000. The genome of *C. cacaofunesta* strain 1593 was generated in the present study (see below) and has been deposited in DDBJ/EMBL/GenBank under the accession code PEJQ00000000.

### Assignment of orthologous coding regions

The phylogenomic analyses assume that the sequences analyzed between species are homologs. The first step in predicting homologous regions was to cluster genes from all genomes of interest using a Markov clustering algorithm implemented in OrthoMCL [121], comprising possible orthologs and paralogs according to their functional groups. Then, all groups composed of only one gene for each species were isolated.

### Sequences of multiple global alignments

Sequences within each orthologous gene families were aligned using the MAFFT 7 software [122] with iterative refinement methods using the weighted sum-of-pair (WSP) and consistency scores (G-INS-i), which implement a pipeline combining the WSP score [123] and the COFFEE-like score [124] to evaluate the consistency between a multiple alignment and pairwise alignments [122]. We used the hmmalign tool of HHMER [125] to analyze the PI-PLC family, which is composed of much diversified proteins. This aligner uses a hidden Markov model (HMM) profile, obtained from a previous alignment of the protein family of interest, to guide the alignment of the domain regions. We downloaded the hmm profile for phospholipase-C in Pfam 27.0 protein database and used the HMMER hmmalign tool.

### Expansion and retraction of gene families

We used the CAFE v3.0 [41] and BadiRate [126] software to analyze changes in gene family size in a way that accounts for phylogenetic history and provides a statistical foundation for evolutionary inferences of expansion and retraction. The CAFE program uses a birth and death process to model gene gain and loss across a phylogenetic tree, which we reconstructed using methods described below. We used the birth and death model in a maximum likelihood statistical approach to infer ancestral states for all tree nodes and calculate a *p*-value. The distribution of family sizes generated under this model provided a basis for assessing the significance of the observed family size differences among taxa.

### Phylogenomic reconstruction

To reconstruct a phylogenetic hypothesis for Sordariomycetes species that presents well-defined branch lengths, we used maximum likelihood methods, implemented in RAxML [127], and Bayesian methods, implemented in MrBayes 3.2 [128], with the preselection of the nucleotide substitution model that best fits the mitochondrial sequences used and keeping length parameter branches without restriction and relaxed relative to the molecular clock. The models were selected using Akaike’s (1974) criteria, as implemented in jModelTest2 [129]. Phylogenetic analysis for *Ceratocystis* species was carried out using a Bayesian analysis, as described below, involving gene beta-tubulin, which was successfully used in a previous broad phylogeny of *Ceratocystis* and related genera [5]. For the *maximum likelihood* analysis, the branch support was estimated using 1000 non-parametric bootstrap repetitions. The resulting tree was visualized and formatted using Figtree 1.3.1 [130]. For the Bayesian analysis, we used two independent rounds of Metropolis-coupled Markov chain Monte Carlo (MCMCMC), with a cold chain and three hot, each analyzed by 1000,000 generations and sampled every 100 generations. The convergence of the chains was determined by inspection through the TRACER 1.5 program [131]. The heating parameters of the chains and the number of generations could be adjusted over the analysis. The list of Sordariomycetes and their genome data bank access numbers are in Additional file 15.

The phylogeny of the PI-PLC gene family was reconstructed using just the maximum likelihood method because the large amount individual genes requires a high computational processing time in Bayesian analysis. The protein alignments guided by HMM profiles had their highly variable ends trimmed using TrimAL [131]. The model of amino acid substitution that fits the data was selected using ProTest [129].

### *Evidence for positive selection in* PI-PLC

In order to estimate the effect of natural selection on PI-PLC genes, the dN/dS ratio was estimated by maximum likelihood using the codon-based model of Goldman and Yang (1994), which was implemented using the codeml program in the PAML 4 package [132]. The divergence among *Ceratocystis* species from their most recent common ancestor was considered to be proportional to the branch sizes in the phylogenetic reconstruction. The enrichment of higher dN/dS rates was tested statistically using the hypergeometric distribution.

### Annotation and search for RIP-like signatures of transposable elements

TEs were identified and annotated from the genome of the fungi *C. cacaofunesta*; also, TEs were identified from *C. fimbriata* CBS 114723 (Bioproject PRJNA67151) and *M. oryzae* (Accession AACU00000000) using the REPET pipeline (http://urgi.versailles.inra.fr/index.php/urgi/Tools/REPET), which was optimized to better annotate nested and fragmented TEs. Repeats were searched with BLASTER [133] for an all-by-all BLASTn genome comparison, clustered with Grouper, RECON, and PILER, and consensuses were built with the MAP Multiple Sequence Alignment program. The consensuses were classified with BLASTER 50 matches, using tBLASTx and BLASTx against the Repbase Update databank, and by identification of structural features such as long terminal repeats and terminal inverted repeats. The \resulting consensuses were used as input for the REPET annotation pipeline part, comprising the TE detection software BLASTER, RepeatMasker, and Censor [133–135]. Localizations of TEs were extracted from the gff3 files, and Blastn was used to find the number and size of TEs in the *C. cacaofunesta* genome. The TE consensuses and their annotated TE copies of the TIR transposon B120, satisfying strict quality criteria (longer than 400 bp in length and at least 80% identical), were aligned using clustalX [136]. This alignment was used for automated analysis of RIP in *C. cacaofunesta* TEs and for estimating dinucleotides using RIPCAL (http://www.sourceforge.net/projects/ripcal) [137]. RIPCAL output provides the numbers of transitions (Ti), transversions (Tv), and dinucleotide targets used in all possible transitions for each TE copy.

### Prediction of structure and homology modeling of C. cacaofunesta PI-PLCs

The experimental data available in Web CD-Search Tool [138–140] was used to annotate function and retrieve domain information for the s111.3 of the expanded PI-PLC gene family of *C. cacaofunesta*. Next, the search for homologous protein with available structural data was performed using BLASTp. Tertiary structure prediction of the protein encoded by gene s111.3 was performed by ViTaMIn4 program using Modeller’s spatial restraints algorithm [141–144]. The homolog phosphatidylinositol-specific phospholipase C proteins from *Listeria monocytogenes* (PDB ID, 1AOD) was used as templates. The loops were refined using Modeller’s ab initio methods [145]. The catalytic site was refined using Dunbrack rotamer library plugin17 for PyMOL19, resulting in 98% of residues in allowed regions of the Ramachandran plot. The structural analysis of these residues indicated they are located in solvent-exposed loop regions away from active site. To confirm the biological relevancy of model, Schrödinger’s Glide [146] was used to predict the binding mode of the substrate inositol in the active site of the modeled protein. The active site of *C. cacaofunesta* PI-PLC was defined as all amino acid residues encompassed within a 20 Å radius sphere centered on the 3D coordinates of the catalytic His58 residue [147, 148]. The docking protocol was repeated 10 times. The default Glide parameters and scoring function were used in docking procedures [45]. Visual inspection was employed to select representative conformation for the ligand.

The sequence alignment of all 76 available genes was conducted with MUSCLE13–15, visualized with JalView217 and compared with gene s111.3 to infer family function [149–151].

### Determination of total cellulases activity in C. cacaofunesta and *C. fimbriata*

*C. cacaofunesta* and *C. fimbriata* were grown on mineral medium [152] containing Avicel® or Avicel® plus xylan from beechwood (2:1) as carbon sources. The cultures were grown during 6 days at 28 °C and 200 rpm. The dosage of total cellulases activity was made according to Ghose (1986) [153] with modifications proposed by Camassola and Dilon (2012) [154]. In summary, 50 μl of the crude supernatants were incubated with a strip of filter paper (1.0 × 0.6 cm) or carboxymethyl cellulose (1%) in 100 μl of sodium acetate buffer, 0.05 M, pH 5.0, during 120 min at 50 °C. The release of reducing sugars was measured by the addition of dinitrosalycilic acid (ADNS) and the reads were done at 540 nm in a spectrophotometer. One unit (U) of enzymatic activity was considered as the amount of enzyme required to release 1 μmol of reducing sugars from the substrate in 1 min under the assay conditions.

## Additional files

Additional file 1: List of predicted genes and annotation of *C. cacaofunesta* and *C. fimbriata*. (XLSX 2372 kb)

Additional file 2: GO classification and KEGG annotation of *C. cacaofunesta* and *C. fimbriata* predicted proteins. (PDF 1084 kb)

Additional file 3: No-hits proteins identified in *C. cacaofunesta* and *C. fimbriata* genomes*. (XLSX 79 kb)*

Additional file 4: OrthoMCL analysis of *C. cacaofunesta* and *C. fimbriata. (XLSX 255 kb)*

Additional file 5: Predicted proteins in CAZy family_GBCAM database_GHs_Cellulose activities. (XLSX 87 kb)

Additional file 6: *C. cacaofunesta* and *C. fimbriata* Secretomes analysis. (XLSX 492 kb)

Additional file 7: Gene family analysis in *Ceratocystis*. (PDF 680 kb)

Additional file 8: *Ceratocystis* genomes comparative analysis*. (PDF 130 kb)*

Additional file 9: TEs in *C. cacaofunesta* and *C. fimbriata* genomes_RIP evidences. (PDF 668 kb)

Additional file 10: *C. cacaofunesta* PI-PLCs Identification_Genomic Distribution_GC content in PI-PLC clusters. (XLSX 26 kb)

Additional file 11: Complete phylogenic analysis of *Ceratocystis* PI-PLCs. (PDF 1635 kb)

Additional file 12: PI-PLCs Molecular modeling. (PDF 1708 kb)

Additional file 13: Blast proteins form True Vascular fungi against C*. cacaofunesta* proteome. (XLSX 11 kb)

Additional file 14: *C. cacaofunesta* GPI-anchored proteins _Effectors CSEP. (XLSX 34 kb)

Additional file 15: Data bank genome access of Sordariomycetes used in comparative studies. (PDF 70 kb)

## Abbreviations

AAs: Auxiliary activities
BLAST: Basic local alignment search tool
Bp: biological process
CAZymes: Carbohydrate-active enzymes
CBMs: Carbohydrate-binding modules
Cc: Cellular component
CDCs: conditionally dispensable chromosomes
CDD: Conserved domains
CEs: Carbohydrate esterases
CMC: Carboxymethyl cellulose
CWC: *Ceratocystis* wilt of cacao
DAG: Diacylglycerol
FPA: Filter paper assay
GHs: Glycoside hydrolases
GO: Gene ontology
GPI: Glycosylphosphatidylinositol
GTs: Glycosyl transferases
In 1,4,5-P3: Inositol 1,4,5-triphosphate
KEGG: Kyoto Encyclopedia of Genes and Genomes
Mf: molecular function
NCBI: National Center for Biotechnology Information
NPP1: *necrosis-inducing protein*
NR: Non-Redundant GenBank Database
ORF: Open read frame
PI 4,5-P2: PI 4,5-bisphosphate
PI 4-P: PI 4-phosphate
PI: Phosphatidylinositol
PI**-**PLCs: Phosphatidylinositol-specific phospholipases-C
PLs: Polysaccharide lyases
RPKM: Reads per kilobase of exons per million
SXM: simulated xylem medium
TEs: transposable elements
