# Supplementary material for: Ceratocystis cacaofunesta genome analysis reveals a large expansion of extracellular phosphatidylinositol-specific phospholipase-C genes (PI-PLC)
Source: BMC Genomics. 2018 Jan 17;19:58. doi: 10.1186/s12864-018-4440-4 (PMC5773145; doi:10.1186/s12864-018-4440-4)
Supplement: Supplementary file 2 — GO classification and KEGG annotation of C. cacaofunesta and C. fimbriata predicted proteins. (PDF 1084 kb) [file 12864_2018_4440_MOESM2_ESM.pdf]

Additional File 2. Functional annotation of total proteome of *C. cacaofunesta* and *C. fimbriata.*

Figure 2. Gene ontology GO and KEGG classification of predicted genes in *C.cacaofunesta* genome A. Biological process (BP). B and C. Distribution of predicted genes involved in metabolic pathway by General and specific KEGG Categories respectively.

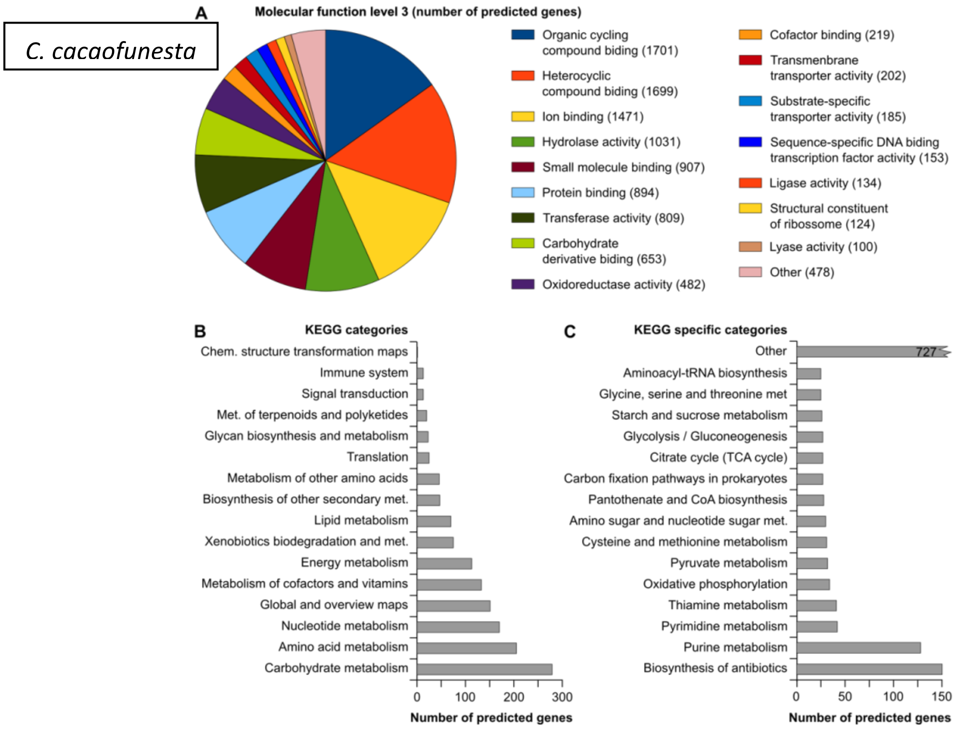

Figure 2. Gene Ontology GO and KEGG classification of predicted genes in *C. cacaofunesta* and *C. fimbriata.* (A). Biological Process (BP). (B) and (C) Distribution of predicted genes involved in pathway by General and Specific KEGG Categories, respectively.

Figure 3. Functional annotation of *C. cacaofunesta* and *C. fimbriata* using GO (Biological Process)

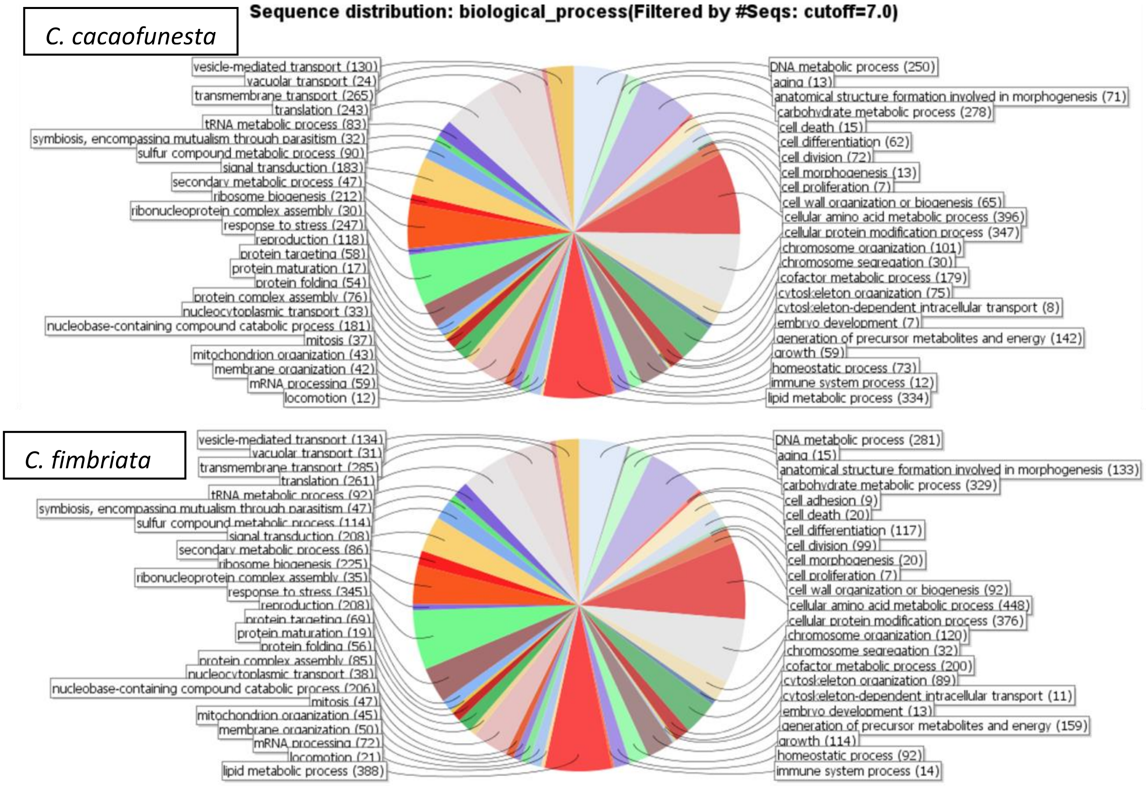

Figure 3. Functional annotation of total *C. cacaofunesta and C. fimbriata* total proteomes – GO/Biological Process, Cutoff 7.

Figure 4. Functional annotation of *C. cacaofunesta* and *C. fimbriata* using GO (Molecular Function).

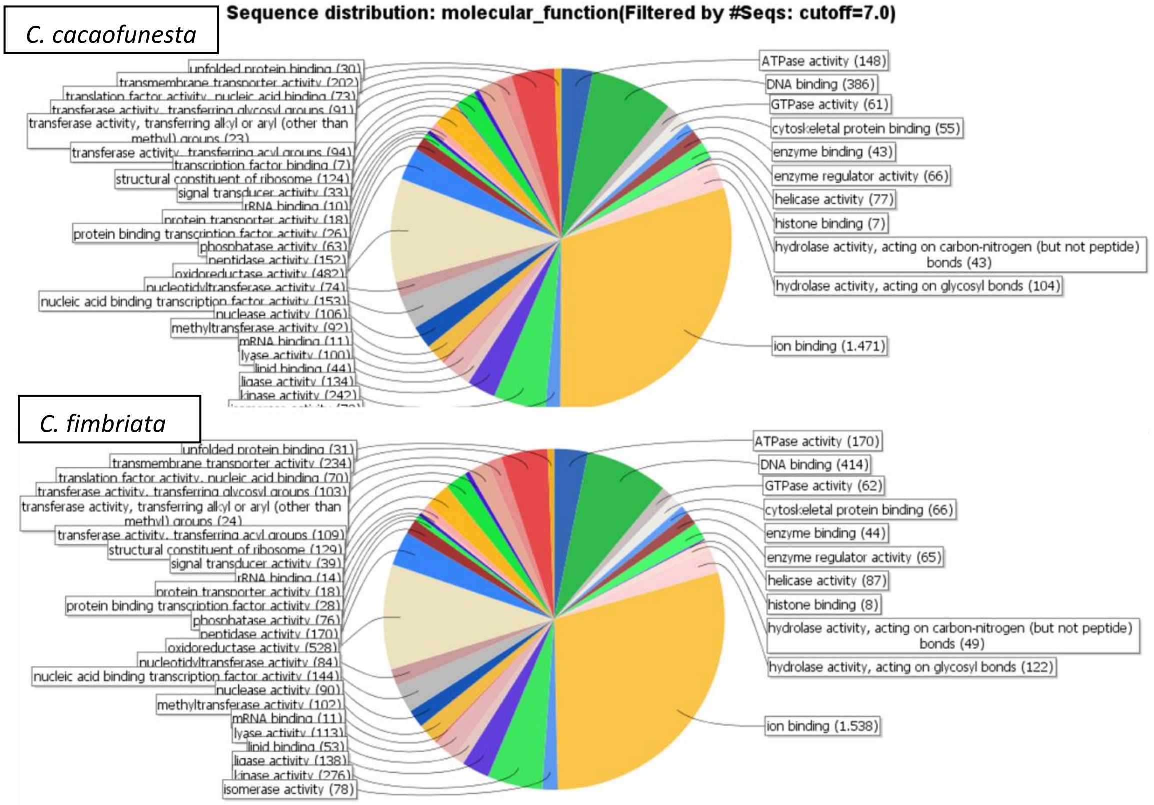

Figure 4. Functional Annotation of *C. cacaofunesta* and *C. fimbriata* total proteomes – GO/Molecular Function, Cut off 7.

Figure 5. Functional annotation of *C. cacaofunesta* and *C. fimbriata* using GO (Biological Cellular component)

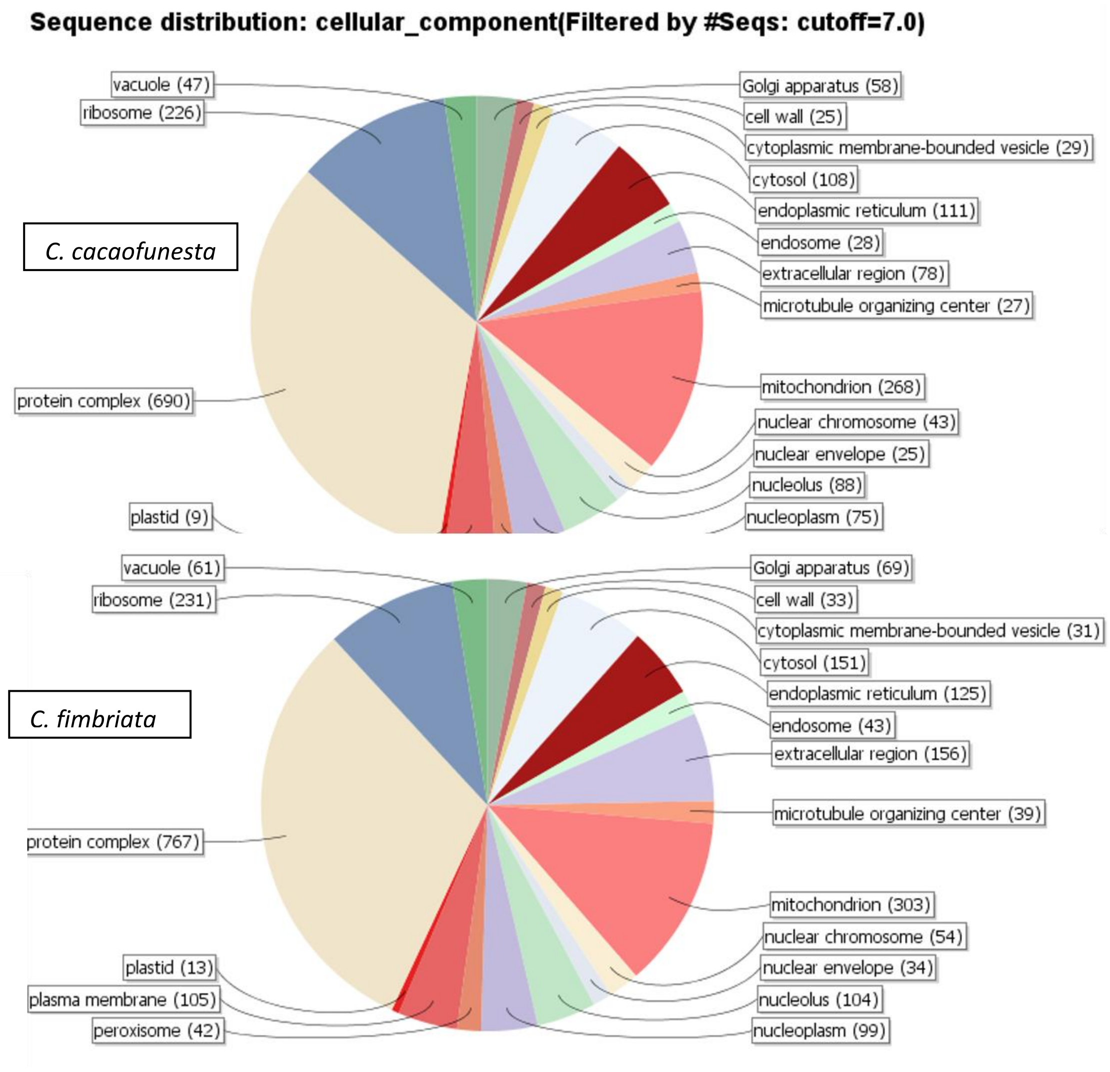

Figure 5. Functional annotation of *C. cacaofunesta* and *C. fimbriata* total proteomes – GO/Cellular Components, Cut off 7.
