# Supplementary material for: Ceratocystis cacaofunesta genome analysis reveals a large expansion of extracellular phosphatidylinositol-specific phospholipase-C genes (PI-PLC)
Source: BMC Genomics. 2018 Jan 17;19:58. doi: 10.1186/s12864-018-4440-4 (PMC5773145; doi:10.1186/s12864-018-4440-4)
Supplement: Supplementary file 7 — Gene family analysis in Ceratocystis. (PDF 680 kb) [file 12864_2018_4440_MOESM7_ESM.pdf]

Additional File 7. Gene family analysis in *Ceratocystis* and other Sordariomycetes.

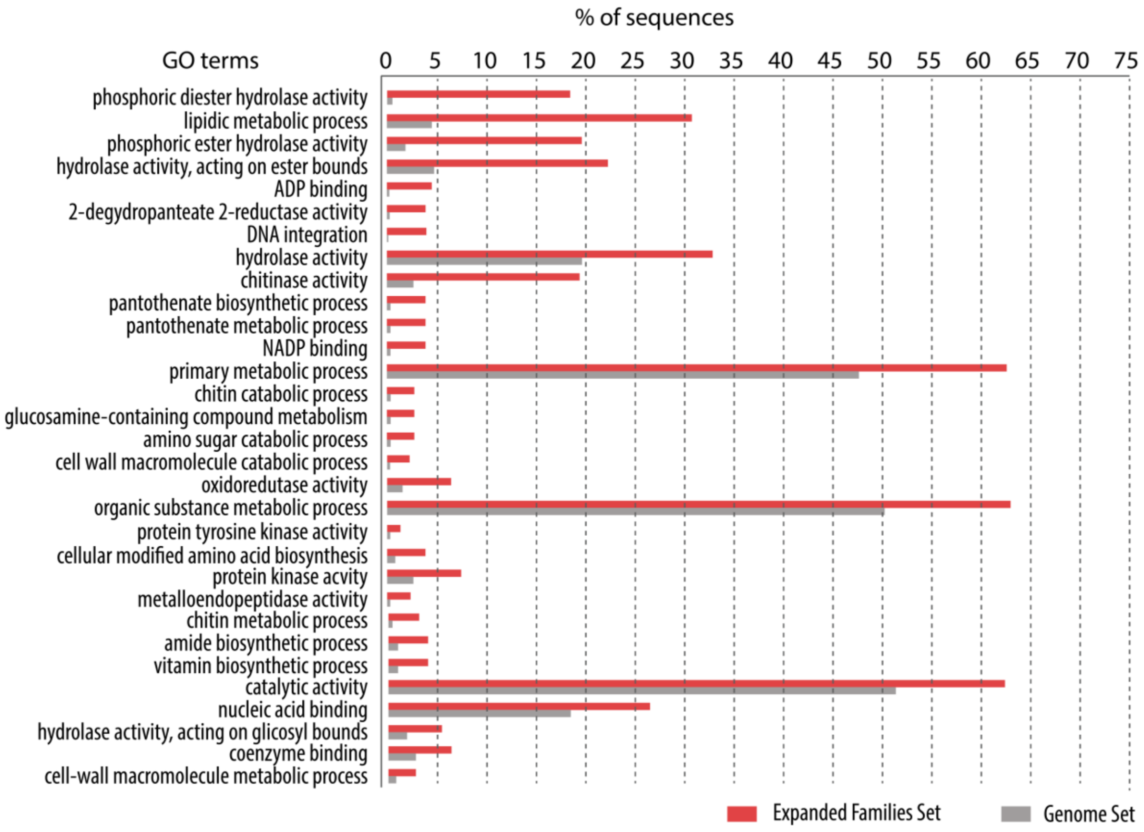

Figure 1. Enrichment of GO terms within the expanded families in *Ceratocystis*.

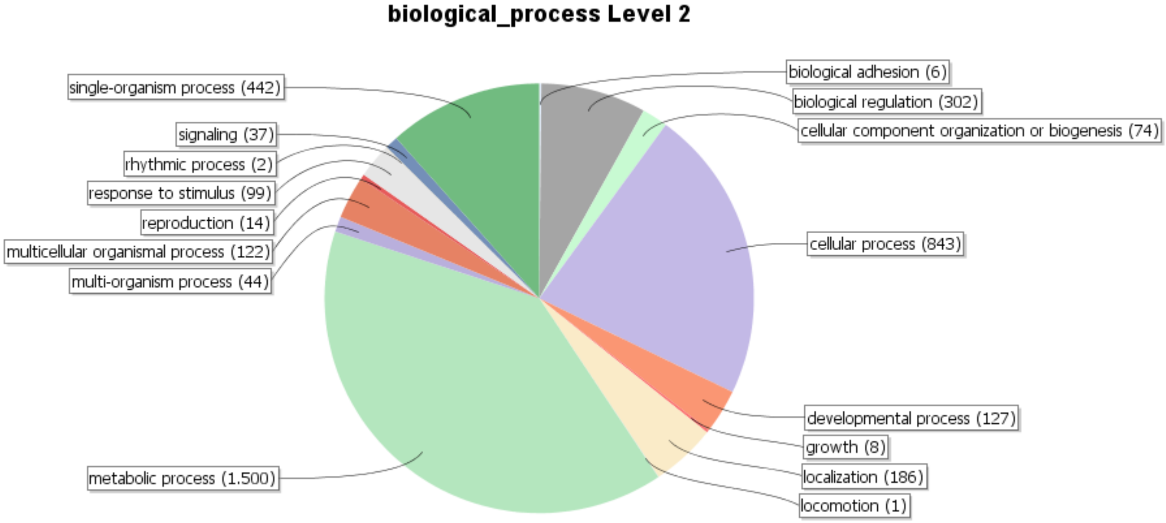

Figure 2. Functional annotation of extinct proteins in *Ceratocystis* using GO/Biological Process.

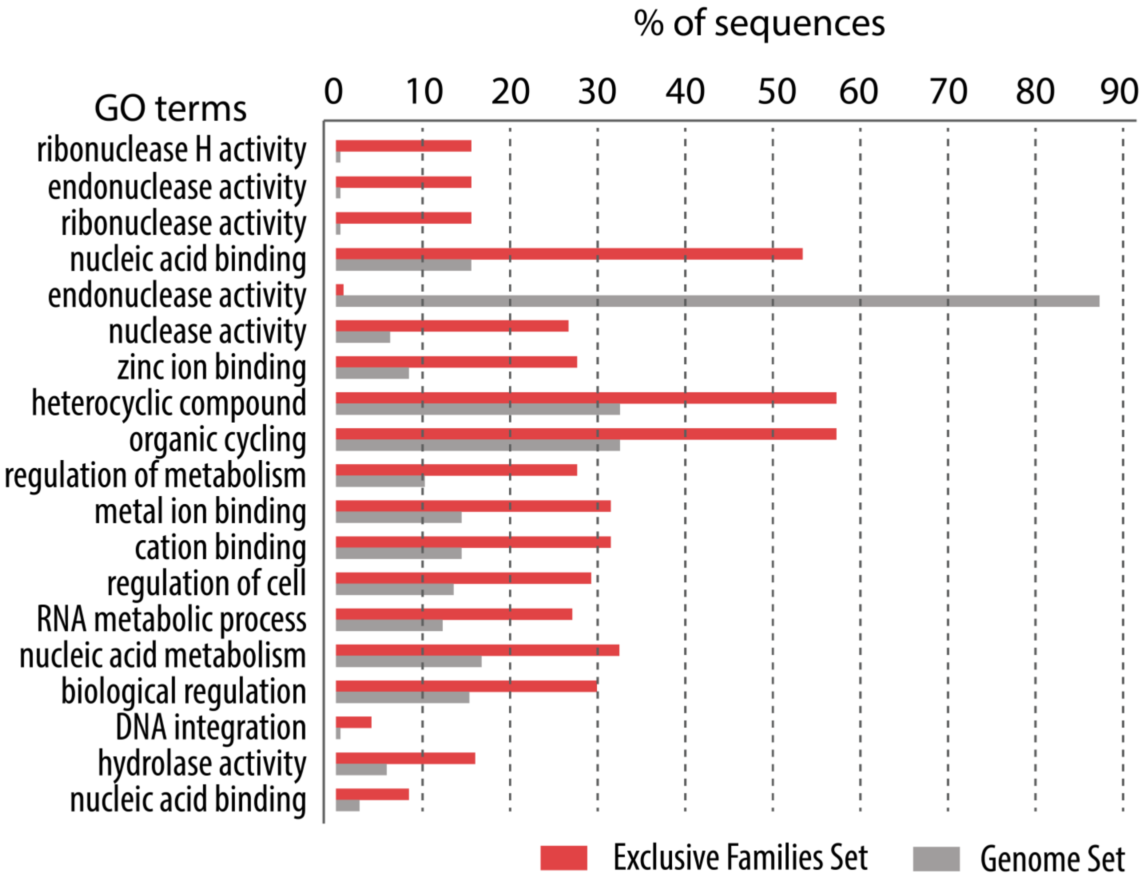

Figure 3. Enrichment of GO terms within the exclusive families of *Ceratocystis*.
