# Supplementary material for: Ceratocystis cacaofunesta genome analysis reveals a large expansion of extracellular phosphatidylinositol-specific phospholipase-C genes (PI-PLC)
Source: BMC Genomics. 2018 Jan 17;19:58. doi: 10.1186/s12864-018-4440-4 (PMC5773145; doi:10.1186/s12864-018-4440-4)
Supplement: Supplementary file 8 — Ceratocystis genomes comparative analysis. (PDF 130 kb) [file 12864_2018_4440_MOESM8_ESM.pdf]

Additional File 8. Comparative analyses within *Ceratocystis* genomes and *Huntiella moniliformis.*

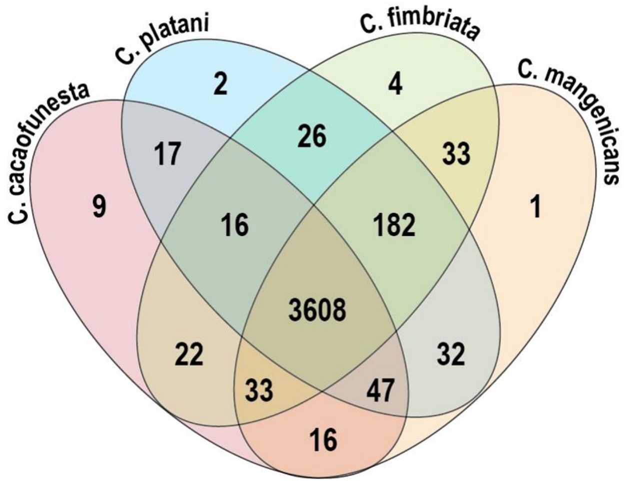

Figure 1. Venn diagram comparing the number of gene clusters shared and exclusive among Ceratocystis species.

Table 1. Genome descriptive statistics for *C. cacaofunesta* compared with other *Ceratocystis* species genomes and *Huntiella moniliformis*.

	Ceratocystis cacaofunest[1]	***Ceratocystis fimbriata***[2]	***Ceratocystis manginecans***[3]	***Ceratocystis platani***[4]	***Huntiella moniliformis***[3]
**Life style**	Necrotrophic	Necrotrophic	Necrotrophic	Necrotrophic	Saprophytic
**Natural hosts**	Cacao	Multiple	Multiple	Platanus	Multiple
**Genome size (Mbp)**	30.50	29.41	31.71	29.18	25.43
**Number of scaffolds**	229	2,641	980	1,213	365
**N50 (kbp)**	386	428	77	77	191
**GC content (%)**	48.1	48.1	47.9	48.2	48.0
**Number of gene models**	7,382	7,200	7,494	5,622	6,832
**Gene density (/Mbp)**	242	245	236	193	269
**Assembly software**	Velvet	Newbler	Velvet	GLC	AbySS
**Genome coverage**	103X	20.0X	22X	655X	38X

1 This study; 2 Wilken et al. 2013; 3 Van der Nest et al. 2014; 4 Belbahri L. 2015.

References:

2. Wilken, P.M. et al., 2013. IMA Genome-F 1: *Ceratocystis fimbriata*: Draft nuclear genome sequence for the plant pathogen, *Ceratocystis fimbriata*. IMA fungus, 4(2), pp.357–8.

3. van der Nest, M.A. et al., 2014. Draft genome sequences of *Diplodia sapinea*, *Ceratocystis manginecans*, and *Ceratocystis moniliformis*. IMA Fungus, 5(1), pp.135–140.
4. Belbahri, L. (2015). Genome sequence of *Ceratocystis platani*, a major pathogen of plane trees. URL http://www. ncbi. nlm. nih. gov/nuccore/814603118.
