# Supplementary material for: Ceratocystis cacaofunesta genome analysis reveals a large expansion of extracellular phosphatidylinositol-specific phospholipase-C genes (PI-PLC)
Source: BMC Genomics. 2018 Jan 17;19:58. doi: 10.1186/s12864-018-4440-4 (PMC5773145; doi:10.1186/s12864-018-4440-4)
Supplement: Supplementary file 9 — TEs in C. cacaofunesta and C. fimbriata genomes_RIP evidences. (PDF 668 kb) [file 12864_2018_4440_MOESM9_ESM.pdf]

Additional File 9. Analysis the TEs of *C. cacaofunesta* genome

RESULTS

C. cacaofunesta genome shows diversity and expansion of TEs

We annotated transposable elements (TE) sequences from three Ascomycota plant-pathogen genomes; *Ceratocystis fimbriata* CBS 114723, *Magnaporthe grisea* 70-15 and *Ceratocystis cacaofunesta*. Different TE content in terms of both; types of elements and amount over these genomes were quantified (Figure 1).

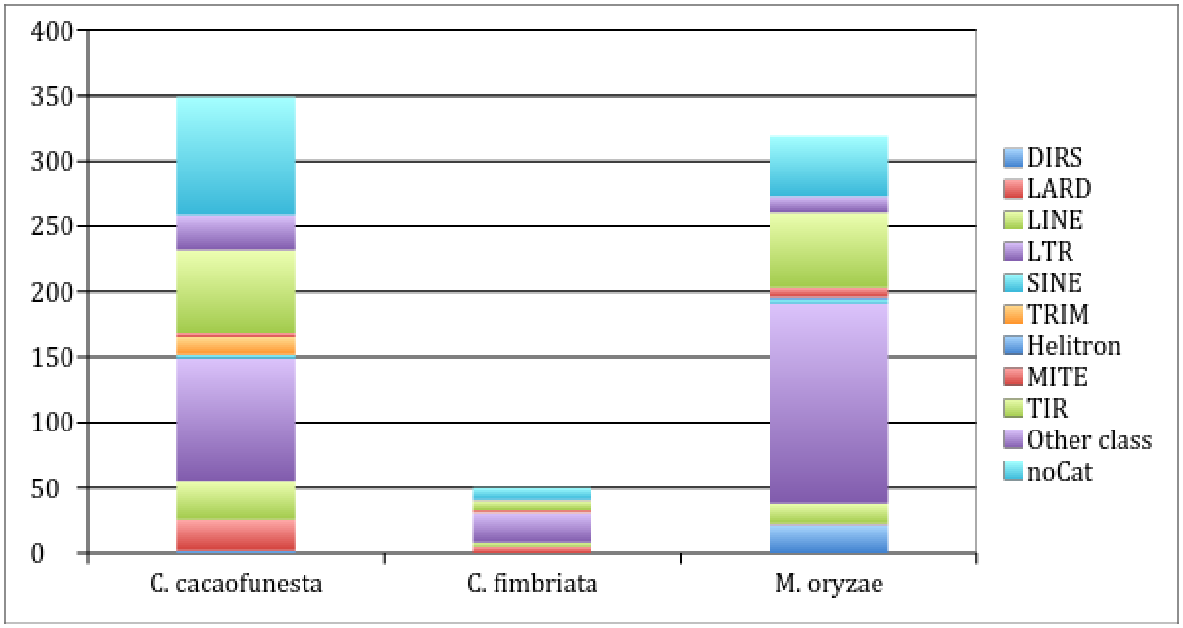

Figure 1. Number and distribution of TEs in three plant-pathogen Ascomycota fungi. Types of TEs.

Genomes from *M. orizae* and *C. cacaofunesta* showed similar content of TE, being *M. orizae* a hemibiotrophic pathogen and *C. cacaofunesta* having a necrothophic lifestyle. However, the necrotrophic *C. fimbriata* showed a low content of TE (0.7%) compared with *C. cacaofunesta*, they having almost the same size of genome (40 Mb, Table 1). The most abundant types of TE in the *C. cacaofunesta* were LTR (Long Terminal Repeats) retrotransposons (27%) and TIR (Terminal Inverted Repeats) DNA transposons (18.3%). Annotation of these TEs showed high identity with the Fot5 and Fonsix6 transposons from

Fusarium oxysporum, and they were broadly located at the scaffolds 3, 10 and 31, next to the PI-PLC clusters.

Table 1 of Additional file 9. Taxonomy of fungal genomes showing genome size and percent of transposable elements adapted from Amselem *et al* 2015. Values with asterisk were calculated in this work using the last version of genome projects.

**Fungi Kingdom**	**Phylum**	**Class Family**	**Lifestyle**	**Strain**	**%TE**	**NCBI BioProject**	**Assambly Size (Mb)**
Botytis cinerea T4	Ascomycota	Leotiomycetes Sclerotiniaceae	Necrotrophic pathogen	T4	0.7	64593	39.5
Botytis cinerea 05.10	Ascomycota	Leotiomycetes Sclerotiniaceae	Necrotrophic pathogen	05.10	2.2	20061	38.8
Blumeria graminis f. sp.hordeï	Ascomycota	Leotiomycetes Erysiphaceae	Obligate biotrophic pathogen	DH14	76.4	28821	120
Ceratocystis cacaofunesta	Ascomycota	Ceratocystidaceae	Necrotrophic pathogen		4.0*		30.4
Caratocystis frimbiata	Ascomycota	Ceratocystidaceae	Necrotrophic pathogen	CBS 114723	0,7	67151	29.4
Leptosphaeria maculans	Ascomycota	Dothideomycetes	Hemibiotrophic pathogen	JN3	33.3	63129	44.9
Magnaporthe oryazae	Ascomycota	Sordariomycetes Magnaporthaceae	Hemibiotrophic pathogen	70-15	4.0*	1433	40.9
Melampsora larici-populina	Basidiomycota	Pucciniomycetes Pucciniaceae	Obligate biotrophic pathogen	98AG31	51.7	46711	101.1
Microbotryum violaceum	Basidiomycota	Pucciniomycetes Mycrobotryaceae	Obligate biotrophic pathogen	P1A1 Lamole	14.1	41281	25.2
Puccinia graminis f. sp. Tritici	Basidiomycota	Pucciniomycetes Pucciniaceae	Obligate biotrophic pathogen	CRL 75-36-700-3	46.4	66375	81.6
Sclerotinia sclerotiorum	Ascomycota	Leotiomycetes Sclerotiniaceae	Necrotrophic pathogen	1980 UF-70	9.5	20263	38.2
Tuber melanosporum	Ascomycota	Pucciniomycetes Mycrobotryaceae	Symbiotic	Mel28	60.1	49017	123.6

Additionally, eleven (3.2%) potential host genes were predicted to be with a high number of repetitions over the genome. Curiously, two of them were located at the high TE density scaffolds 6 and 31, next to the Fot5 transposon. Scaffolds 31 and 6 were the most diverse in terms of types of TEs. They had twelve different classes of TEs (Figure 2). Annotation of these host genes showed to be enzymes related with Phospholipase C pathway and Cytochrome b complex.

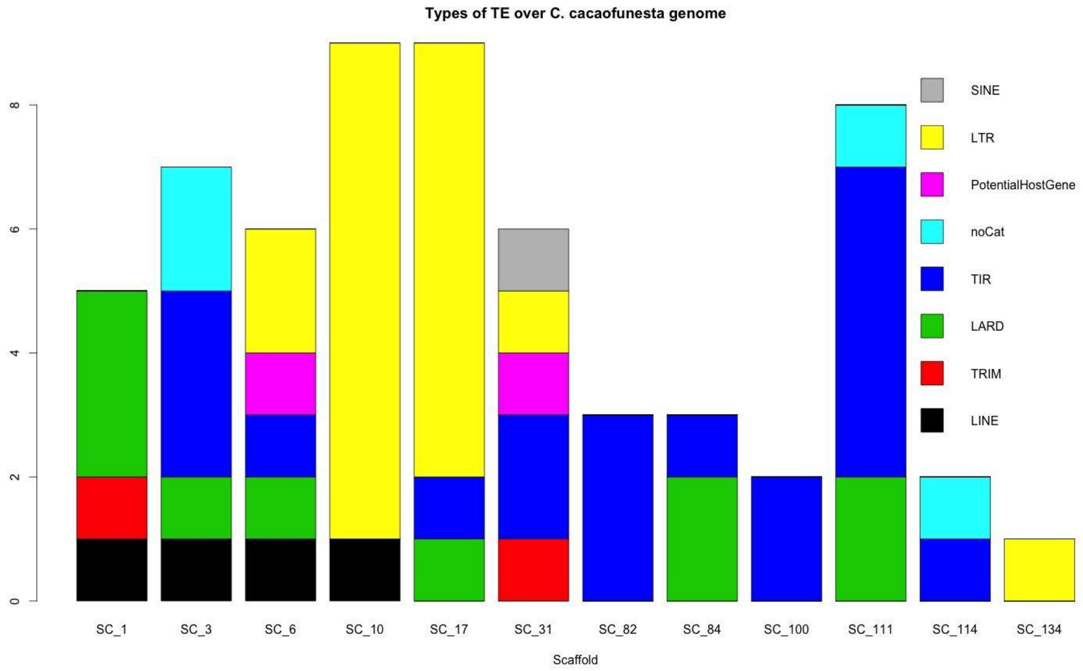

Figure 2. Distribution of types of TEs in the most density scaffold over the *C. cacaofunesta* genome

Additionally, we used TEs copies of the TIR transposon B120, similar to Fot5 located along of the scaffolds 31 and 84 for estimating of transition and transversions mediated by RIP mechanism. A clear CpA → TpA dinucleotide bias was detected for this transposon (Figure 3). We found a ratio of transitions (Ti) over transversions (Tv) of 2.3 fold more than a given random mutation ratio that it must be of 0.5. It indicating that RIP mechanism can be inactivating extra copies in the genome, however it result must be verified experimentally.

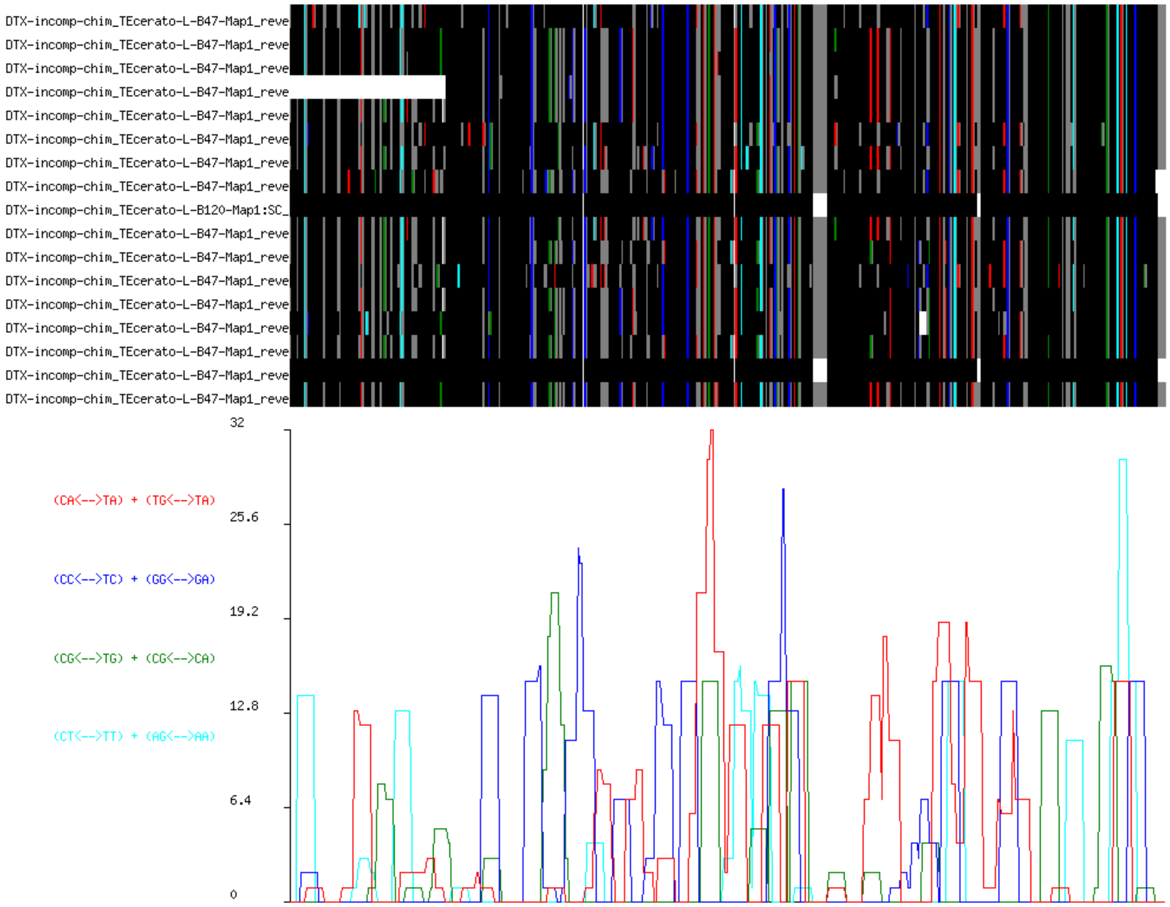

Figure 3. Analyses of RIP mechanism in *C. cacaofunesta* TEs.

DISCUSSION

In order to characterize expanded families in the *C. cacaofunesta* genome, we performed an analysis of transposable elements (TEs) using REPET pipeline for the three genomes of *C. cacaofunesta*, *C. fimbriata* CBS 114723 and *Magnaporthe grisea* 70-15. We found massive TE expansions over the *C. cacaofunesta* genome, indicating that it can be playing a significant role in genome structure, dynamic and evolution (Coghlan et al 2005). TEs cause chromosomal rearrangements and it plays an important role in adaptation and evolution of the fungi, providing novel DNA sequences, gene duplication, gene loss and inactivation (Beimont et al, 2010). It has been largely reported in other pathogens Ascomycota fungi such as *M. graminicola* (Amselem et al 2015) and it shows to accelerate

the evolution of genes that affect pathogenicity and host range (Manning et al 2013, Fudal et al 2009). Genome size expansion due to repeat elements was also reported in the oomycete *Phytophthora infestans* compared to *P. ramorum*. This alteration was mainly mediated by TEs expansion, creating gene-rich islands separated by vast expanses of repetitive sequences (Haas et al., 2009). Other example of genome structure alteration due TEs expansion was documented in the *M. graminicola* genome where a single-copy DNA methyltransferase gene was duplicated into a subtelomeric region and then amplified among the telomeres to a dozen copies, all of which were subsequently recognized by the repeat-induced point mutation (RIP) machinery and inactivated, including the original copy (Dhillon et al., 2010). Amselem et al. (2011) found similar patterns studying the genomes of neurotrophic fungal pathogens *Sclerotinia sclerotiorum* and *Botrytis cinerea,* where higher content and new composition of TEs were discovered in *S. sclerotiorum* compared to *B. cinerea* and they related with host-range adaptation. Finally, in the *Verticillium dahliae* LS.17 genome, was found notable TE clustering in chromosomes 3 and 4 containing "hot-spots" of TE insertions, harboring around 20% of the transposons and duplicated copies of genes coding chitinase and phospholipase enzymes (Amyotte et al. 2012).

Different composition of TEs between *C. fimbriata* and *C. cacaofunesta* are due to the recent invasion of a few families of class II TIR DNA and LINE TEs in *C. cacaofunesta*. A subfamily of TIR TEs, similar to the Fos5 transposon of *Fusarium oxysporum* are composed of almost identical copies dispersed in the genome and next to them were found gene expansion of Phospholipases enzymes. *Fot* family has been identified, with a much higher diversification and representation in the genomes of *F. oxysporum* and *N. haematococ*ca (Drufresne et al. 2011). Additionally, Schimidt et al. (2013), searching novel virulence genes in *F. oxyporum* located the Fos5 downstream of SIX (Secreted in Xylem) genes and it associated with MITE (miniature inverted-repeat transposable element) on pathogenecity chromosome. Thus, we know now that the transposons are frequently present in gene-rich locations in plant-pathogen fungal genomes, and shows a putative sequence duplication event involving genes encoding a chitinase and a phospholipase for host adaptation (Klosterman et al. 2011).

Transposable elements impact on Whole-Genome architecture and transcriptional profiles, due to their inherent ability to amplify (Castanera, 2016). However, defense mechanisms exist in the genome to minimize the numbers of TEs. One such mechanism that is specific to fungi is Repeat-Induced Point mutation (RIP) (Idnurm and Howlett, 2003). One major consequence of RIP involves genome G:C content, because RIP increases the A:T content of mutated TE copies. As a result, when TEs are clustered in large blocks, the RIP-mediated mutation of C:G to A:T generates A:T rich isochores, as observed in Lmac (Rouxel et al, 2011). Repetitive sequences in *M. graminicola* exhibited a CpA dinucleotide bias, the same as seen in *Neurospora crassa* (Cambareri et al 1989). In *N. crassa*, RIP increased the occurrence of termination codons TAG and TAA (Singer et al 1995). Gypsy_17 Phospholipase A2 Expression of orthologous genes displaying TE insertion in *P. ostreatus* genome (Castanera, 2016).

METHODOLOGY

Annotation and search for RIP-like signatures of Transposable Elements

TEs were identified and annotated from the genome of the fungi *C. cacaofunesta* (this work), *C. fimbriata* CBS 114723 (Bioproject PRJNA67151) and *M. oryzae* (Accession AACU00000000), using the „REPET" pipeline (http://urgi.versailles.inra.fr/index.php/urgi/Tools/REPET), optimized to better annotate nested and fragmented TEs. Repeats were searched with BLASTER for an all-by-all BLASTn genome comparison, clustered with Grouper, RECON and PILER, and consensuses built with the MAP multiple sequence alignment program. Consensuses were classified with BLASTER 50 matches, using tBLASTx and BLASTx against the Repbase Update databank and by identification of structural features such as long terminal repeats, terminal inverted repeats. Resulting consensuses were used as input for the REPET annotation pipeline part, comprising the TE detection software BLASTER, RepeatMasker and Censor, and the satellite detection softwares RepeatMasker. Localizations of TEs were extracted from the gff3 files and Blastn was used to find the number and size of TEs in the *C. cacaofunesta* genome. TE consensus with their annotated TE copies of the TIR transposon B120, satisfying strict quality criteria (longer than 400 bp in length and at least

80% identical) were aligned using clustalX (Larkin, et al 2017). This alignment was used for automated analysis of RIP in *C. cacaofunesta* TEs and estimating di-nucleotide using RIPCAL (http://www.sourceforge.net/projects/ ripcal) (Hane JK and Oliver RP, 2008). RIPCAL output provides the number of transitions (Ti), transversions (Tv) and dinucleotide targets used in all possible transitions for each TE copy.

REFERENCES

- Amselem J, Cuomo CA, van Kan JAL, Viaud M, Benito EP, et al. (2011) Genomic Analysis of the Necrotrophic Fungal Pathogens *Sclerotinia sclerotiorum* and *Botrytis cinerea*. PLoS Genet 7(8): e1002230. doi:10.1371/journal.pgen.1002230
- Amyotte SG, Tan X, Pennerman K, et al. Transposable elements in phytopathogenic *Verticillium spp*.: insights into genome evolution and inter- and intra-specific diversification. BMC Genomics. 2012;13:314. doi:10.1186/1471-2164-13-314.
- Biemont C. A brief history of the status of transposable elements: from junk DNA to major players in evolution. Genetics. 2010;186:1085–93.
- Cambareri EB, Jensen BC, Schabtach E, Selker EU: Repeat-induced G-C to A-T mutations in *Neurospora*. Science 1989, 244(4912):1571–1575.
- Castanera R, López-Varas L, Borgognone A, LaButti K, Lapidus A, Schmutz J, et al. (2016) Transposable Elements versus the Fungal Genome: Impact on Whole-Genome Architecture and Transcriptional Profiles. PLoS Genet 12(6): e1006108. doi:10.1371/journal.pgen.1006108
- Coghlan A, Eichler EE, Oliver SG, Paterson AH, Stein L. Chromosome evolution in eukaryotes: a multi-kingdom perspective. Trends Genet. 2005;21:673–82.
- Dhillon B, Cavaletto JR, Wood KV, Goodwin SB: Accidental amplification and inactivation of a methyltransferase gene eliminates cytosine methylation in *Mycosphaerella graminicola*. Genetics 2010, 186(1):67–77.
- Dufresne, M., Lespinet, O., Daboussi, M. Hua-Van A. Genome-Wide Comparative Analysis of *pogo*-Like Transposable Elements in Different *Fusarium* Species. J Mol Evol (2011) 73: 230. doi:10.1007/s00239-011-9472-1
- Fudal I, Ross S, Brun H, Besnard AL, Ermel M, Kuhn ML, et al. Repeat-induced point mutation (RIP) as an alternative mechanism of evolution toward virulence in *Leptosphaeria maculans*. Mol Plant Microbe Interact. 2009;22:932–41.
- Hane JK, Oliver RP. RIPCAL: a tool for alignment-based analysis of repeat-induced point mutations in fungal genomic sequences. BMC Bioinformatics. 2008;9:478.
- Haas BJ, Kamoun S, Zody MC, Jiang RH, Handsaker RE, Cano LM, Grabherr M, Kodira CD, Raffaele S, Torto-Alalibo T, et al. 2009. Genome sequence and analysis of the Irish potato famine pathogen Phytophthora infestans. Nature 461: 393–398.
- Idnurm Alexander and Howlett Barbara J. Analysis of loss of pathogenicity mutants reveals that repeat-induced point mutations can occur in the *Dothideomycete Leptosphaeria maculans*, Fungal Genetics and Biology, Volume 39, Issue 1, June 2003, Pages 31-37, ISSN 1087-1845, http://dx.doi.org/10.1016/S1087-1845(02)00588-1.

- Joelle Amselem, Marc-Henri Lebrun and Hadi Quesneville. Whole genome comparative analysis of transposable elements provides new insight into mechanisms of their inactivation in fungal genomes. BMC Genomics (2015) 16:141. DOI 10.1186/s12864-015-1347-1
- Klosterman SJ, Subbarao KV, Kang S, Veronese P, Gold SE, et al. (2011) Comparative Genomics Yields Insights into Niche Adaptation of Plant Vascular Wilt Pathogens. PLoS Pathog 7(7): e1002137. doi:10.1371/journal.ppat.1002137
- Larkin MA, Blackshields G, Brown NP, Chenna R, McGettigan PA, McWilliam H, Valentin F, Wallace IM, Wilm A, Lopez R, Thompson JD, Gibson TJ, Higgins DG: Clustal W and Clustal X version 2.0. Bioinformatics 2007, 23(21):2947–2948.
- Manning VA, Pandelova I, Dhillon B, Wilhelm LJ, Goodwin SB, Berlin AM, et al. Comparative genomics of a plant-pathogenic fungus, *Pyrenophora tritici-repentis*, reveals transduplication and the impact of repeat elements on pathogenicity and population divergence. G3 (Bethesda). 2013;3:41–63.
- Schmidt SM, Houterman PM, Schreiver I, et al. MITEs in the promoters of effector genes allow prediction of novel virulence genes in *Fusarium oxysporum*. *BMC Genomics*. 2013;14:119. doi:10.1186/1471-2164-14-119.
- Singer MJ, Marcotte BA, Selker EU: DNA methylation associated with repeat-induced point mutation in *Neurospora crassa*. Mol Cell Biol 1995, 15(10):5586–5597.
- Rouxel T, Grandaubert J, Hane JK, Hoede C, van de Wouw AP, Couloux A, et al. Effector diversification within compartments of the *Leptosphaeria maculans* genome affected by Repeat-Induced Point mutations. Nat Commun. 2011;2:202.
