# Supplementary material for: Ceratocystis cacaofunesta genome analysis reveals a large expansion of extracellular phosphatidylinositol-specific phospholipase-C genes (PI-PLC)
Source: BMC Genomics. 2018 Jan 17;19:58. doi: 10.1186/s12864-018-4440-4 (PMC5773145; doi:10.1186/s12864-018-4440-4)
Supplement: Supplementary file 11 — Complete phylogenic analysis of Ceratocystis PI-PLCs. (PDF 1635 kb) [file 12864_2018_4440_MOESM11_ESM.pdf]

Additional File 11. Complete phylogenic analysis of *Ceratocystis* PI-PLCs.

Legend. Complement of figure 7 phylogeny of PI-PLC gene family in *Ceratocystis* species. Fifteen clusters were defined being one ancestral, and the other 14 (A-N) equally related in a star-like branch. This figure shows cluster B to N. Posterior probabilities of the Bayesian Inference are above branches. A table in the right compiles information of the proteins. Grey circles on phylogeny nodes indicate groups of othologous genes.

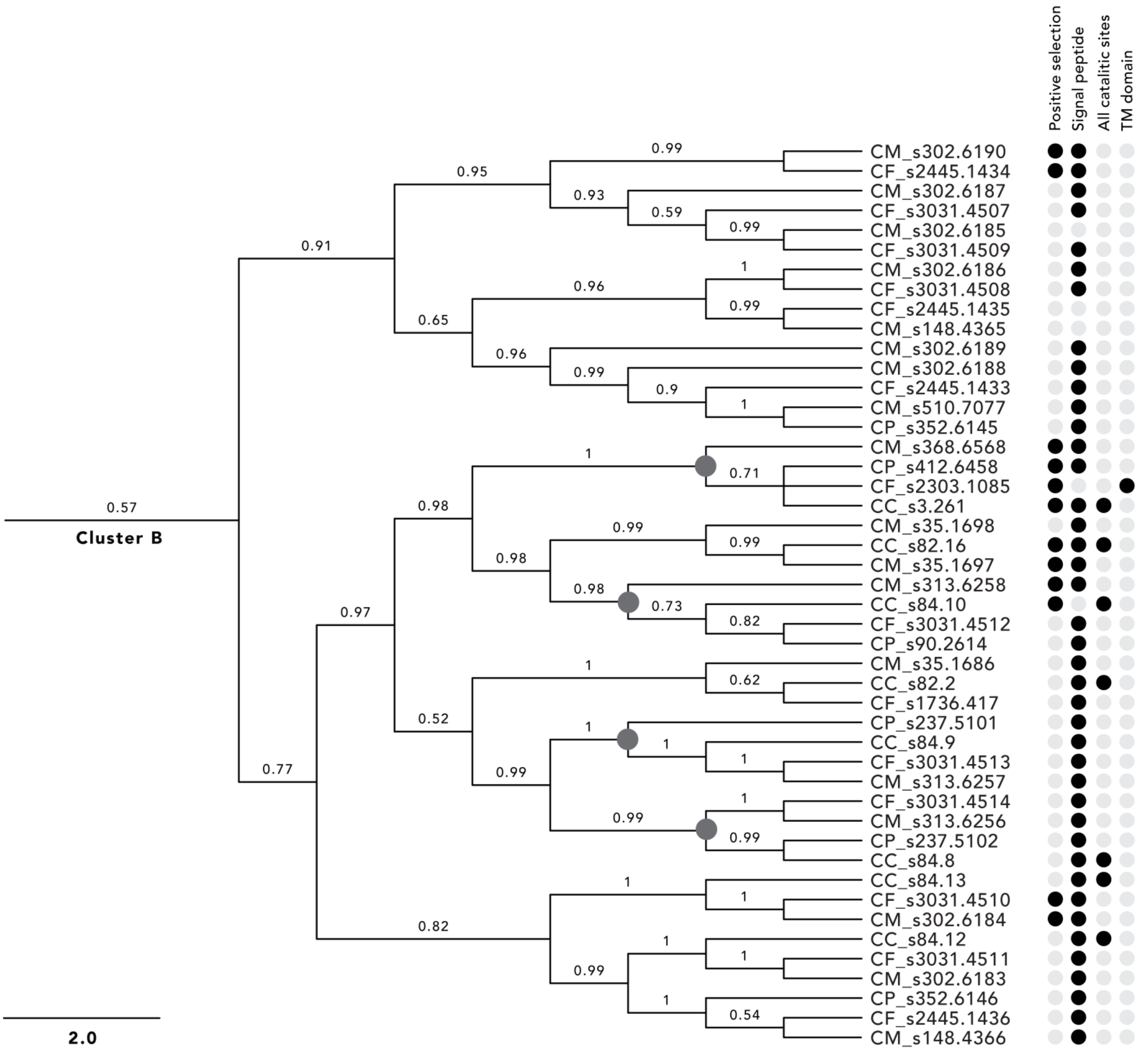

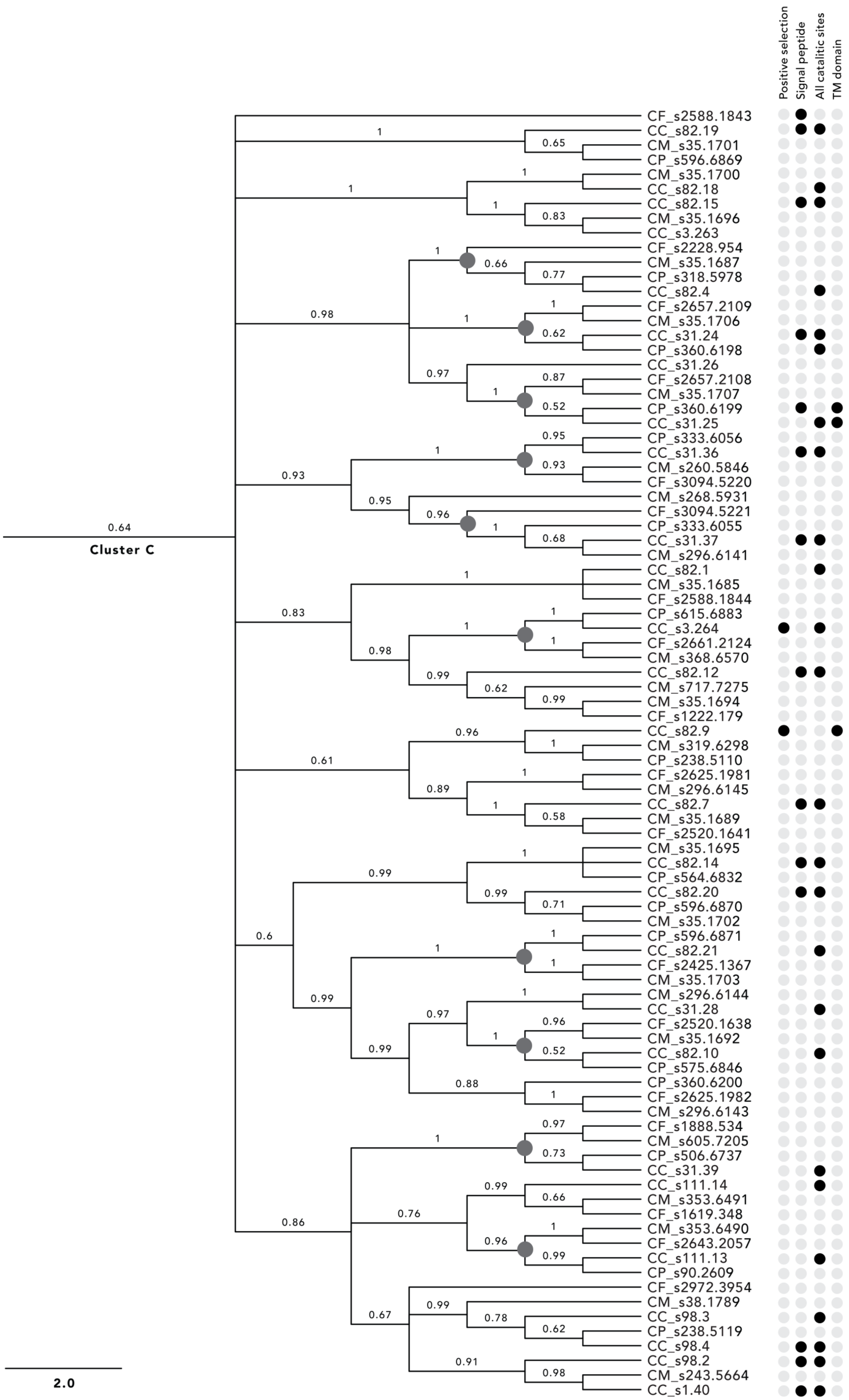

Positive selection
Signal peptide
All catalitic sites
TM domain
Cluster C
2.0
CF_s2588.1843
CC_s82.19
CM_s35.1701
CP_s596.6869
CM_s35.1700
CC_s82.18
CC_s82.15
CM_s35.1696
CC_s3.263
CF_s2228.954
CM_s35.1687
CP_s318.5978
CC_s82.4
CF_s2657.2109
CM_s35.1706
CC_s31.24
CP_s360.6198
CC_s31.26
CF_s2657.2108
CM_s35.1707
CP_s360.6199
CC_s31.25
CP_s333.6056
CC_s31.36
CM_s260.5846
CF_s3094.5220
CM_s268.5931
CF_s3094.5221
CP_s333.6055
CC_s31.37
CM_s296.6141
CC_s82.1
CM_s35.1685
CF_s2588.1844
CP_s615.6883
CC_s3.264
CF_s2661.2124
CM_s368.6570
CC_s82.12
CM_s717.7275
CM_s35.1694
CF_s1222.179
CC_s82.9
CM_s319.6298
CP_s238.5110
CF_s2625.1981
CM_s296.6145
CC_s82.7
CM_s35.1689
CF_s2520.1641
CM_s35.1695
CC_s82.14
CP_s564.6832
CC_s82.20
CP_s596.6870
CM_s35.1702
CP_s596.6871
CC_s82.21
CF_s2425.1367
CM_s35.1703
CM_s296.6144
CC_s31.28
CF_s2520.1638
CM_s35.1692
CC_s82.10
CP_s575.6846
CP_s360.6200
CF_s2625.1982
CM_s296.6143
CF_s1888.534
CM_s605.7205
CP_s506.6737
CC_s31.39
CC_s111.14
CM_s353.6491
CF_s1619.348
CM_s353.6490
CF_s2643.2057
CC_s111.13
CP_s90.2609
CF_s2972.3954
CM_s38.1789
CC_s98.3
CP_s238.5119
CC_s98.4
CC_s98.2
CM_s243.5664
CC_s1.40

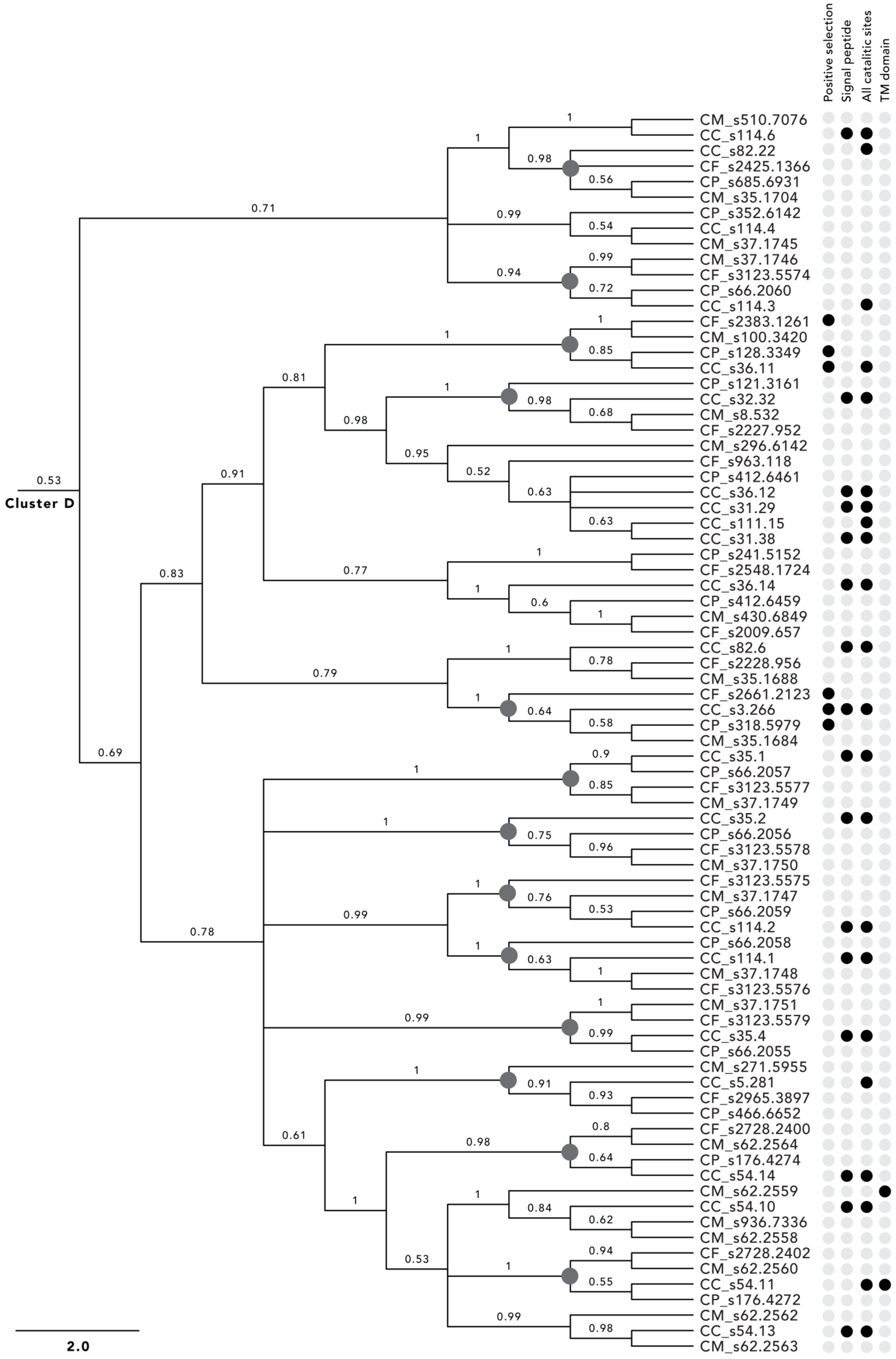
Positive selection
Signal peptide
All catalitic sites
TM domain
Cluster D
CM_s510.7076
CC_s114.6
CC_s82.22
CF_s2425.1366
CP_s685.6931
CM_s35.1704
CP_s352.6142
CC_s114.4
CM_s37.1745
CM_s37.1746
CF_s3123.5574
CP_s66.2060
CC_s114.3
CF_s2383.1261
CM_s100.3420
CP_s128.3349
CC_s36.11
CP_s121.3161
CC_s32.32
CM_s8.532
CF_s2227.952
CM_s296.6142
CF_s963.118
CP_s412.6461
CC_s36.12
CC_s31.29
CC_s111.15
CC_s31.38
CP_s241.5152
CF_s2548.1724
CC_s36.14
CP_s412.6459
CM_s430.6849
CF_s2009.657
CC_s82.6
CF_s2228.956
CM_s35.1688
CF_s2661.2123
CC_s3.266
CP_s318.5979
CM_s35.1684
CC_s35.1
CP_s66.2057
CF_s3123.5577
CM_s37.1749
CC_s35.2
CP_s66.2056
CF_s3123.5578
CM_s37.1750
CF_s3123.5575
CM_s37.1747
CP_s66.2059
CC_s114.2
CP_s66.2058
CC_s114.1
CM_s37.1748
CF_s3123.5576
CM_s37.1751
CF_s3123.5579
CC_s35.4
CP_s66.2055
CM_s271.5955
CC_s5.281
CF_s2965.3897
CP_s466.6652
CF_s2728.2400
CM_s62.2564
CP_s176.4274
CC_s54.14
CM_s62.2559
CC_s54.10
CM_s936.7336
CM_s62.2558
CF_s2728.2402
CM_s62.2560
CC_s54.11
CP_s176.4272
CM_s62.2562
CC_s54.13
CM_s62.2563
2.0

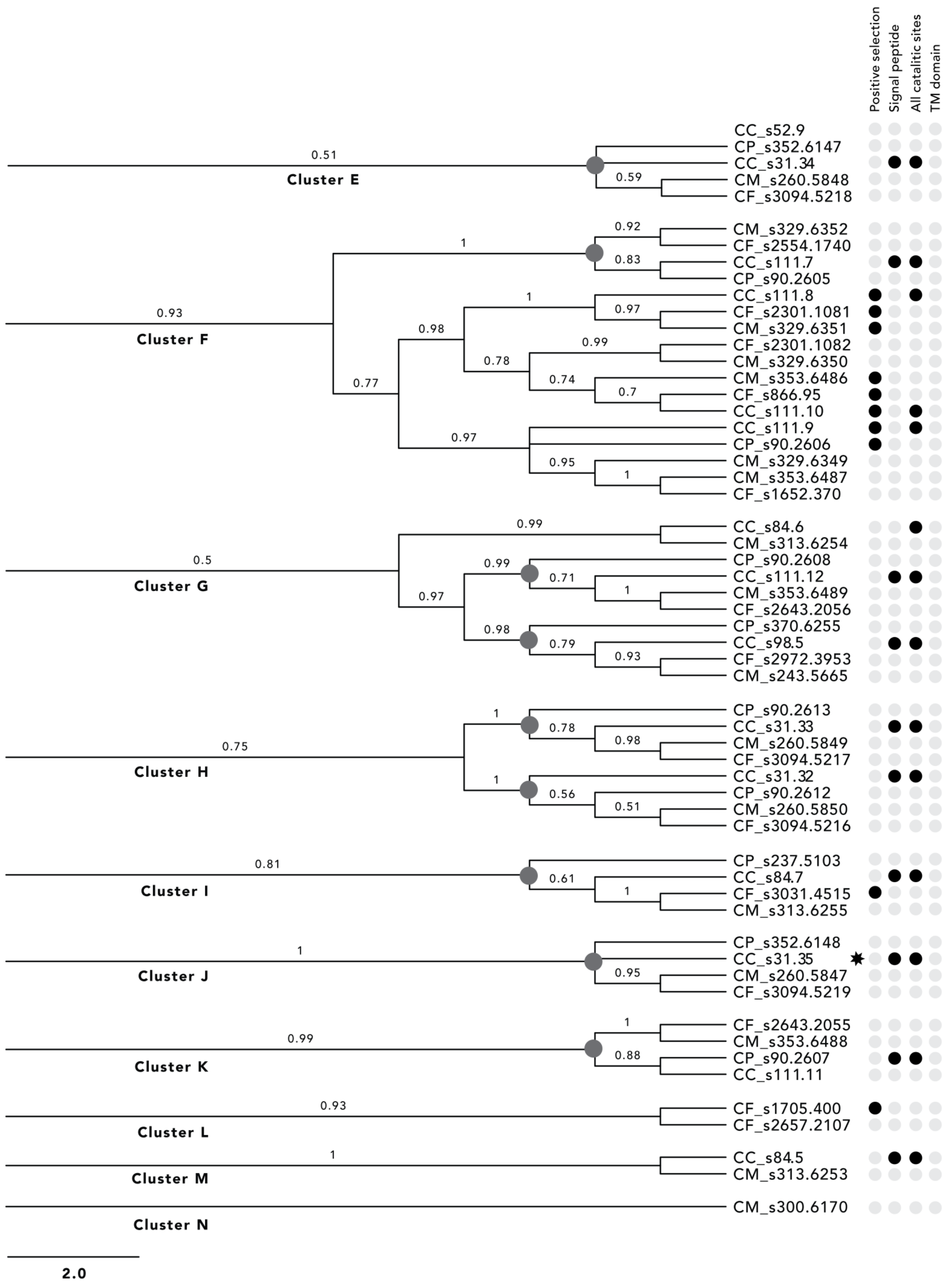
Positive selection
Signal peptide
All catalitic sites
TM domain
Cluster E
CC_s52.9
CP_s352.6147
CC_s31.34
CM_s260.5848
CF_s3094.5218
Cluster F
CM_s329.6352
CF_s2554.1740
CC_s111.7
CP_s90.2605
CC_s111.8
CF_s2301.1081
CM_s329.6351
CF_s2301.1082
CM_s329.6350
CM_s353.6486
CF_s866.95
CC_s111.10
CC_s111.9
CP_s90.2606
CM_s329.6349
CM_s353.6487
CF_s1652.370
Cluster G
CC_s84.6
CM_s313.6254
CP_s90.2608
CC_s111.12
CM_s353.6489
CF_s2643.2056
CP_s370.6255
CC_s98.5
CF_s2972.3953
CM_s243.5665
Cluster H
CP_s90.2613
CC_s31.33
CM_s260.5849
CF_s3094.5217
CC_s31.32
CP_s90.2612
CM_s260.5850
CF_s3094.5216
Cluster I
CP_s237.5103
CC_s84.7
CF_s3031.4515
CM_s313.6255
Cluster J
CP_s352.6148
CC_s31.35
CM_s260.5847
CF_s3094.5219
Cluster K
CF_s2643.2055
CM_s353.6488
CP_s90.2607
CC_s111.11
Cluster L
CF_s1705.400
CF_s2657.2107
Cluster M
CC_s84.5
CM_s313.6253
Cluster N
CM_s300.6170
2.0
