# Supplementary material for: Ceratocystis cacaofunesta genome analysis reveals a large expansion of extracellular phosphatidylinositol-specific phospholipase-C genes (PI-PLC)
Source: BMC Genomics. 2018 Jan 17;19:58. doi: 10.1186/s12864-018-4440-4 (PMC5773145; doi:10.1186/s12864-018-4440-4)
Supplement: Supplementary file 12 — PI-PLCs Molecular modeling. (PDF 1708 kb) [file 12864_2018_4440_MOESM12_ESM.pdf]

Additional File 12. Structural model of *C. cacaofunesta* PI-PLC family

Table 1. Comparative table between active site residues of the most similar crystallographic model (PDB ID 1AOD) and modeled protein (Gene CC619). It is highlighted in bold the existing mutations. The score refers to the value given for substitution among the residues in the BLOSUM62 scoring matrix.

Modelagem	Cristalográfico	Pontuação	Modelagem	Cristalográfico	Pontuação
His58	His	15	***Gln140***	***Lys***	***1***
Asn59	***Glu***	***1***	***Asp199***	***Arg***	***-1***
Thr78	Thr	11	***Ser227***	***Thr***	***1***
Asp93	Asp	11	***His257***	***Phe***	***-2***
Thr95	***Arg***	***-1***	***Thr259***	***Ser***	***1***
Asn107	Asn	6	Ser261	Ser	10
Arg138	Arg	17			

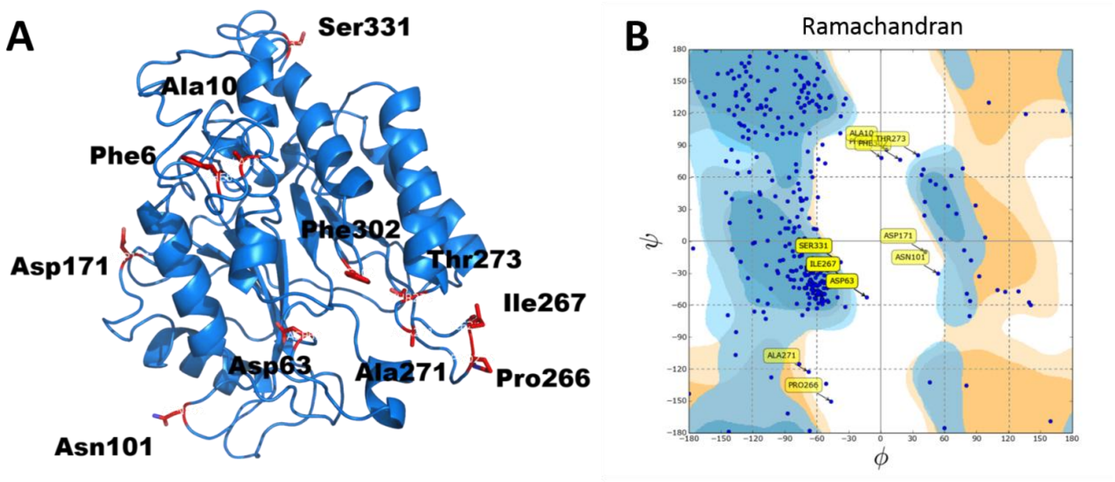

Regiões permitidas = **91,4%**; Regiões adicionais = **6,5**%; Regiões proibidas = **2,1**%

Figura 1. (A) Position of residues outside the allowed region in the Ramachandran diagram. (B) Ramachandran diagram for the constructed model. The detached residues are in regions distal to the catalytic site and account for 2.1% of the total residues.

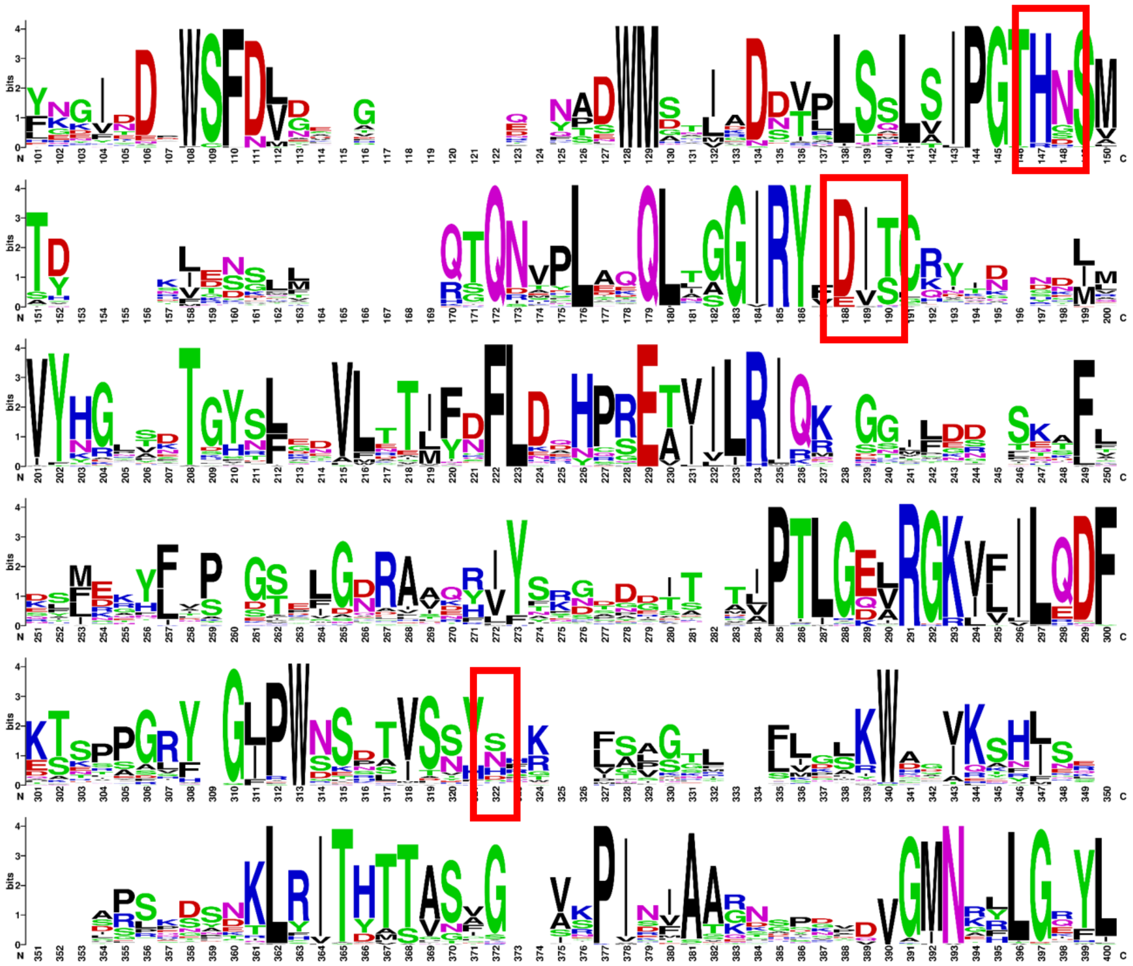

Figura 2. Logotype representation of sequence alignment of 75 genes from the phosphoinositol-specific phospholipase family of C. cacaofunesta. In this scheme, the larger the size of the logo representing an amino acid residue, the greater its occurrence in that position within the alignment. Very poorly conserved positions present many residues in the same position and very conserved positions, few residues. The consensus sequence represents the highest occurrence residue for each of the positions. The scheme shows only the portion of the alignment containing the catalytic residues. In particular, catalytic residues.

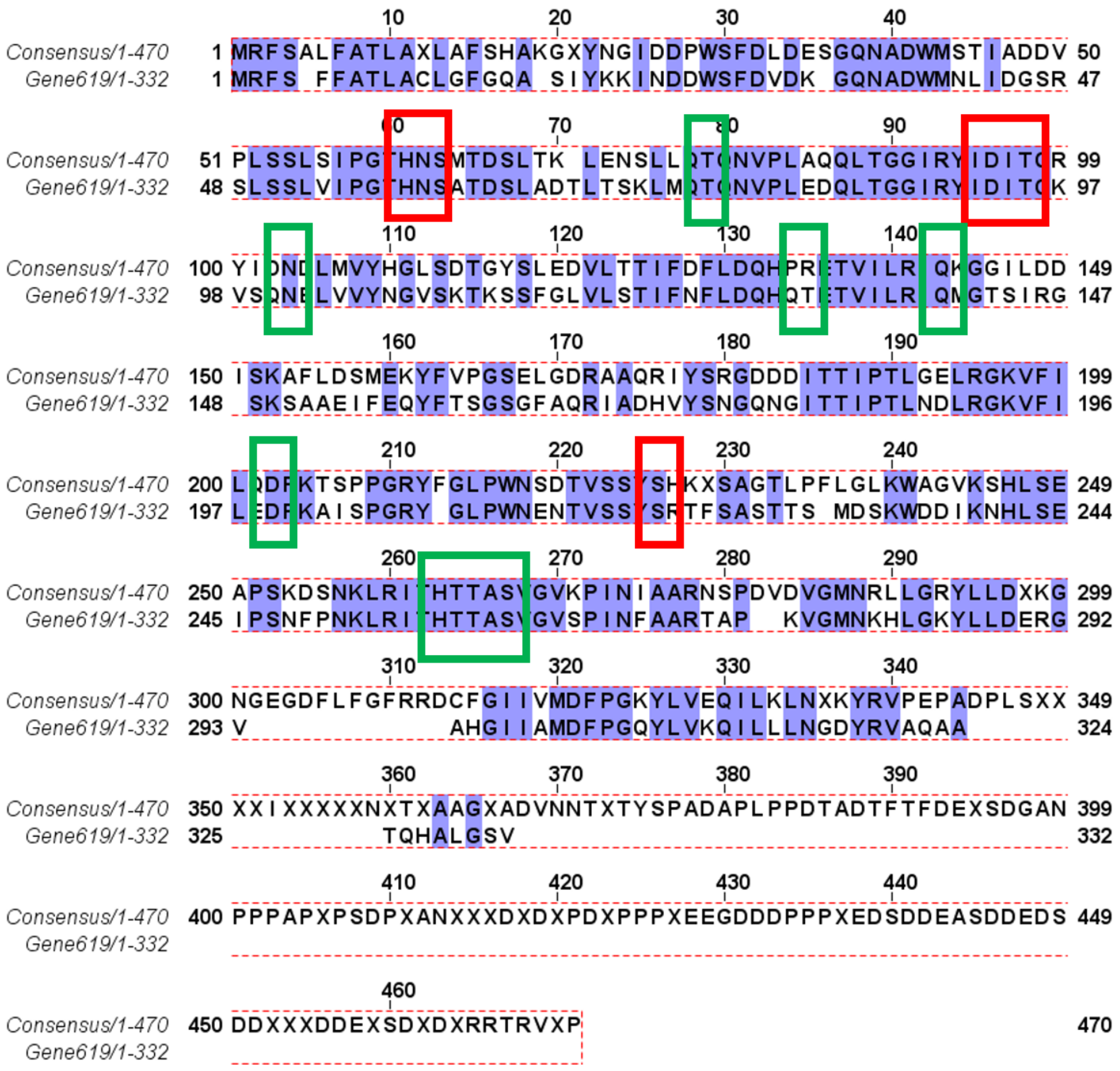

Figura 3. Alignment of the CC619 gene sequence and the consensus sequence of the remainder members of the PI-PLC family. The red markings represent the residues corresponding to those identified as catalytic in the crystallographic structure and those in green, the residues of the active site.

```
sp|P09194|PHLC_TRYBB        RPYVE------------------------------------YPYARLRSVALKSIW----
sp|P45723|PLC_STAAE         NRMGGTYIKSGYGADTSGIQ---------------------WADNATFETKINNGS----
tr|Q81WS7|Q81WS7_BACAN      KRYSGSNESGGYNN----FY---------------------WPDNETFTTTVNQNV----
sp|P14262|PLC_BACCE         KRYSGSNEPGGYNN----FY---------------------WPDNETFTTTVNQNA----
sp|P08954|PLC_BACTU         KRYSGSNESGGYNN----FY---------------------WPDNETFTTTVNQNV----
model1                      EDFKAIS-PGRYG----------------------------LPWNENTVSSYSRTF----
sp|O13433|PLC1_CANAX        QKYCSND--NGWNKESLNEYLLSSYSTPYREITQTQTNYYDYPLNEYFISSSHNTYLTGR
                                                                     .
```

Figura 4. Alignment of basterial PI-PLC and *C. cacaofunesta* PI-PLC in the region of the beta-strand. In green, the presence of 8 amino acids responsible for the formation of the beta-strand.

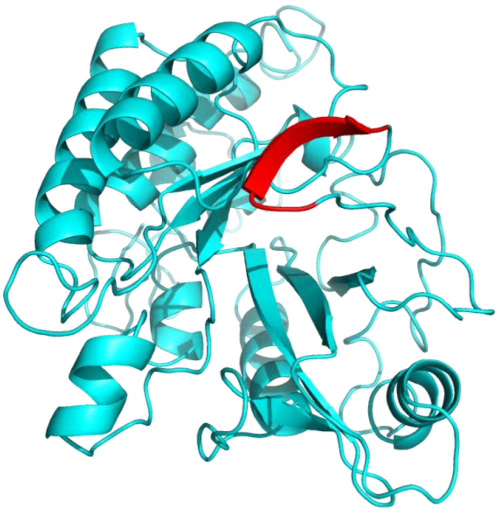

Figura 5. Model of the PI-PLC (gene CC619). In red, the beta- strand.
