# Supplementary material for: Ceratocystis cacaofunesta genome analysis reveals a large expansion of extracellular phosphatidylinositol-specific phospholipase-C genes (PI-PLC)
Source: BMC Genomics. 2018 Jan 17;19:58. doi: 10.1186/s12864-018-4440-4 (PMC5773145; doi:10.1186/s12864-018-4440-4)
Supplement: Supplementary file 15 — Data bank genome access of Sordariomycetes used in comparative studies. (PDF 70 kb) [file 12864_2018_4440_MOESM15_ESM.pdf]

Additional File 15.

Table 1. Identification and characterization of the Sordariomycete genomes used in comparative analyses. Species in bold are plant pathogens.

References cited in Table 1.

Wilken, P. M., Steenkamp, E. T., Wingfield, M. J., De Beer, Z. W., & Wingfield, B. D. (2013). IMA Genome-F 1: Ceratocystis fimbriata Draft nuclear genome sequence for the plant pathogen, Ceratocystis fimbriata. IMA fungus, 4(2), 357-358.

Bock, T., Chen, W. H., Ori, A., Malik, N., Silva-Martin, N., Huerta-Cepas, J., ... & Kirkpatrick, J. (2014). An integrated approach for genome annotation of the eukaryotic thermophile Chaetomium thermophilum. Nucleic acids research, 42(22), 13525-13533.

Zheng, P., Xia, Y., Xiao, G., Xiong, C., Hu, X., Zhang, S., ... & Zhao, G. P. (2011). Genome sequence of the insect pathogenic fungus Cordyceps militaris, a valued traditional Chinese medicine. Genome biology, 12(11), R116.

Cuomo, C. A., Güldener, U., Xu, J. R., Trail, F., Turgeon, B. G., Di Pietro, A., ... & Adam, G. (2007). The Fusarium graminearum genome reveals a link between localized polymorphism and pathogen specialization. Science, 317(5843), 1400-1402.

Cuomo, C. A., Untereiner, W. A., Ma, L. J., Grabherr, M., & Birren, B. W. (2015). Draft genome sequence of the cellulolytic fungus Chaetomium globosum. Genome announcements, 3(1), e00021-15.Cuomo, C. A., Untereiner, W. A., Ma, L. J., Grabherr, M., & Birren, B. W. (2015). Draft genome sequence of the cellulolytic fungus Chaetomium globosum. Genome announcements, 3(1), e00021-15.

Ma, L. J., Van Der Does, H. C., Borkovich, K. A., Coleman, J. J., Daboussi, M. J., Di Pietro, A., ... & Houterman, P. M. (2010). Comparative genomics reveals mobile pathogenicity chromosomes in Fusarium. Nature, 464(7287), 367.

Coleman, J. J., Rounsley, S. D., Rodriguez-Carres, M., Kuo, A., Wasmann, C. C., Grimwood, J., ... & Schwartz, D. C. (2009). The genome of Nectria haematococca: contribution of supernumerary chromosomes to gene expansion. PLoS genetics, 5(8), e1000618.

Randhir, R. J., & Hanau, R. M. (1997). Size and complexity of the nuclear genome of Colletotrichum graminicola. Applied and environmental microbiology, 63(10), 4001-4004.

Dean, R. A., Talbot, N. J., Ebbole, D. J., & Farman, M. L. (2005). The genome sequence of the rice blast fungus Magnaporthe grisea. Nature, 434(7036), 980.

Gao, Q., Jin, K., Ying, S. H., Zhang, Y., Xiao, G., Shang, Y., ... & Peng, G. (2011). Genome sequencing and comparative transcriptomics of the model entomopathogenic fungi Metarhizium anisopliae and M. acridum. PLoS genetics, 7(1), e1001264.

Galagan, J. E., Calvo, S. E., Borkovich, K. A., Selker, E. U., & Read, N. D. (2003). The genome sequence of the filamentous fungus Neurospora crassa. Nature, 422(6934), 859.

Ellison, C. E., Stajich, J. E., Jacobson, D. J., Natvig, D. O., Lapidus, A., Foster, B., ... & Taylor, J. W. (2011). Massive changes in genome architecture accompany the transition to self-fertility in the filamentous fungus Neurospora tetrasperma. Genetics, 189(1), 55-69.

Haridas, S., Wang, Y., Lim, L., Alamouti, S. M., Jackman, S., Docking, R., ... & Breuil, C. (2013). The genome and transcriptome of the pine saprophyte Ophiostoma piceae, and a comparison with the bark beetle-associated pine pathogen Grosmannia clavigera. BMC genomics, 14(1), 373.

Espagne, E., Lespinet, O., Malagnac, F., Da Silva, C., Jaillon, O., Porcel, B. M., ... & Anthouard, V. (2008). The genome sequence of the model ascomycete fungus Podospora anserina. Genome biology, 9(5), R77.

Berka, R. M., Grigoriev, I. V., Otillar, R., Salamov, A., Grimwood, J., Reid, I., ... & Henrissat, B. (2011). Comparative genomic analysis of the thermophilic biomass-degrading fungi Myceliophthora thermophila and Thielavia terrestris. Nature biotechnology, 29(10), 922-927.

Kubicek, C. P., Herrera-Estrella, A., Seidl-Seiboth, V., Martinez, D. A., Druzhinina, I. S., Thon, M., ... & Mukherjee, M. (2011). Comparative genome sequence analysis underscores mycoparasitism as the ancestral life style of Trichoderma. Genome biology, 12(4), R40.

Martinez, D., Berka, R. M., Henrissat, B., Saloheimo, M., Arvas, M., Baker, S. E., ... & Danchin, E. G. (2008). Genome sequencing and analysis of the biomass-degrading fungus Trichoderma reesei (syn. Hypocrea jecorina). Nature biotechnology, 26(5), 553.

Klosterman, S. J., Subbarao, K. V., Kang, S., Veronese, P., Gold, S. E., Thomma, B. P., ... & Garcia-Pedrajas, M. D. (2011). Comparative genomics yields insights into niche adaptation of plant vascular wilt pathogens. PLoS pathogens, 7(7), e1002137.

	Size	%GC total	Genes	Gene density	Mean protein length (aa)	GenBank accession	Ref.
Ceratocystis cacaofunesta	**30.500.000**	**48,1**	**7269**	**4195,9**	**542**		**this paper**
Ceratocystis fimbriata	29.400.000	48,3	7609	3863,8	517	GCA_000389695.2	Wilken *et al.* 2013
Chaetomium globosum	34.900.000	55,6	11124	3137,4	493	GCA_000143365.1	Cuomo *et al.* 2015
Chaetomium thermophilum	28.322.806	52,6	7475	3789,0	547	GCA_000221225.1	Bock *et al.* 2014
Cordyceps militaris	32.210.000	51,4	9651	3337,5	504	GCA_000225605.1	Zheng *et al.* 2011
Fusarium graminearum	36.446.046	48,4	13289	2742,9	446	GCA_000599445.1	Cuomo *et al.* 2007
Fusarium oxysporum	61.357.934	48,4	20897	2936,3	407	GCA_000259975.2	Ma *et al.* 2010
Fusarium solani	51.150.000	50,8	15707	3256,5	480	GCA_002215905.1	Coleman *et al.* 2009
Fusarium verticillioides	41.776.161	48,7	15865	2633,5	420	GCA_000149555.1	Ma *et al.* 2010
Glomerella graminicola	51.604.658	49,2	11942	4263,1	466	GCA_000149035.1	Randhir *et al.* 1997
Magnaporthe grisea	41.695.457	51,6	11054	3772,4	481	GCA_001548815.1	Dean *et al.* 2005
Metarhizium acridum	39.422.329	49,9	9849	4002,7	494	GCA_000187405.1	Gao *et al.* 2011
Metarhizium anisopliae	39.145.269	51,4	10583	3698,9	507	GCA_000814975.1	Gao *et al.* 2011
Neurospora crassa	40.416.174	48,9	10785	3805,3	438	GCA_000786625.1	Galagan *et al.* 2003
Neurospora tetrasperma	39.146.357	49,4	10380	3766,9	468	GCA_000213175.1	Ellison *et al.* 2011
Ophiostoma piceae	33.000.000	52,8	8884	3714,5	467	GCA_000410735.1	Haridas *et al.* 2013
Podospora anserina	35.010.595	52,2	10588	3306,6	507	GCA_000226545.1	Espagne *et al.* 2008
Thielavia terrestris	36.912.256	54,7	9813	3761,3	464	GCA_000226115.1	Berka et al. 2011
Trichoderma atroviride	36.100.000	49,8	11816	3043,1	456	GCA_000963795.1	Kubicek *et al.* 2011
Trichoderma reesei	34.100.000	52,0	9129	3729,6	493	GCA_000167675.2	Martinez *et al.* 2008
Trichoderma virens	39.022.666	49,2	12427	3138,3	471	GCA_000170995.2	Kubicek *et al.* 2011
Verticilium alfalfae	30.299.901	56,1	10187	3222,7	438	GCA_000150825.1	Klosterman *et al.* 2011
Verticillium dahliae	33.828.453	55,9	10948	3090,1	474	GCA_000150675.2	Klosterman et al. 2011
